# Allele-Specific Genome-wide Profiling in Human Primary Erythroblasts Reveal Replication Program Organization

**DOI:** 10.1371/journal.pgen.1004319

**Published:** 2014-05-01

**Authors:** Rituparna Mukhopadhyay, Julien Lajugie, Nicolas Fourel, Ari Selzer, Michael Schizas, Boris Bartholdy, Jessica Mar, Chii Mei Lin, Melvenia M. Martin, Michael Ryan, Mirit I. Aladjem, Eric E. Bouhassira

**Affiliations:** 1 Department of Cell Biology, Albert Einstein College of Medicine, Bronx, New York, United States of America; 2 Department of Systems and Computational Biology, Albert Einstein College of Medicine, Bronx, New York, United States of America; 3 Laboratory of Molecular Pharmacology, Center for Cancer Research, National Cancer Institute, Bethesda, Maryland, United States of America; Friedrich Miescher Institute for Biomedical Research, Switzerland

## Abstract

We have developed a new approach to characterize allele-specific timing of DNA replication genome-wide in human primary basophilic erythroblasts. We show that the two chromosome homologs replicate at the same time in about 88% of the genome and that large structural variants are preferentially associated with asynchronous replication. We identified about 600 megabase-sized asynchronously replicated domains in two tested individuals. The longest asynchronously replicated domains are enriched in imprinted genes suggesting that structural variants and parental imprinting are two causes of replication asynchrony in the human genome. Biased chromosome X inactivation in one of the two individuals tested was another source of detectable replication asynchrony. Analysis of high-resolution TimEX profiles revealed small variations termed timing ripples, which were undetected in previous, lower resolution analyses. Timing ripples reflect highly reproducible, variations of the timing of replication in the 100 kb-range that exist within the well-characterized megabase-sized replication timing domains. These ripples correspond to clusters of origins of replication that we detected using novel nascent strands DNA profiling methods. Analysis of the distribution of replication origins revealed dramatic differences in initiation of replication frequencies during S phase and a strong association, in both synchronous and asynchronous regions, between origins of replication and three genomic features: G-quadruplexes, CpG Islands and transcription start sites. The frequency of initiation in asynchronous regions was similar in the two homologs. Asynchronous regions were richer in origins of replication than synchronous regions.

## Author Summary

DNA replication in mammalian cells proceeds according to a distinct order. Genes that are expressed tend to replicate before genes that are not expressed. We report here that we have developed a method to measure the timing of replication of the maternal and paternal chromosomes separately. We found that the paternal and maternal chromosomes replicate at exactly the same time in the large majority of the genome and that the 12% of the genome that replicated asynchronously was enriched in imprinted genes and in structural variants. Previous experiments have shown that chromosomes could be divided into replication timing domains that are a few hundred thousand to a few megabases in size. We show here that these domains can be divided into sub-domains defined by ripples in the timing profile. These ripples corresponded to clusters of origins of replication. Finally, we show that the frequency of initiation in asynchronous regions was similar in the two homologs.

## Introduction

DNA replication in eukaryotes starts at DNA sequences termed origins of replication. The timing of DNA replication within the S-phase of the cell cycle depends on the distribution and the relative order of activation of those origins. Eukaryotic DNA replication is mediated by the sequential formation of a pre-Replication Complex (preRC) in the G1 phase of the cell cycle, starting with the binding of the origin recognition complex (ORC) to DNA and followed by the binding of additional licensing proteins that lead to activation of replication origins in S phase [1]–[3]. Although in some loci origins are constitutive and initiate replication each cell cycle, in most loci origin usage is very flexible and there are many more pre-RC complexes than origins that actually initiate replication [1], [4], [5].

The timing of initiation events and the precise chromosomal location of the origins of replication seem to be determined independently [6]. The timing of replication might be determined at the level of large chromatin domains early in G1, while origin selection, a process that determines which potential replication origin will initiate replication in a particular cell cycle, would occur later in G1. Several reports have shown that origin selection is also influenced by events taking place in mitosis [7]–[9]. The flexibility in origin usage results in the detection of replication initiation zones, which are DNA regions in which many origins can be mapped and therefore where initiation seems very frequent. Origins in initiation zones are used alternatively, apparently stochastically, in the different cells of a homogeneous cell population [10]. This flexibility might be necessary to allow multicellular organisms to replicate their genomes in cells with different chromatin structure or after DNA damage [1].

Several models have been proposed to explain the general organization of the replication program. These models differ in two basic assumptions. First, one set of models assumes that the frequency of initiation from each particular origin might be regulated independently of the time of replication during the cell cycle [11], whereas another set of models assumes that the replication time and initiation frequency are co-regulated [12], [13] Second, these models also differ in whether they postulate the existence of mechanisms that coordinate initiation from multiple origins of replication (See [14] for a detailed discussion).

Computer simulations and analyses at the single molecule level have led to the independent origin hypothesis, which proposes that origins initiate replication stochastically, independently from neighboring origins [11]. According to this model, the sole determinant of the timing of replication would be the efficiency of individual origins rather than a mechanism that directly regulates the time at which origins start replication. Simulations based on the independent origin hypothesis can model replication very successfully in budding yeast [11] and have been applied effectively to explain the regulation of the IgH locus in mammals [14]. In the case of mammalian cells, the next-in-line and the domino-cascade models [12], [13] have been proposed to take into consideration the idea that neighboring origins can be co-regulated.

On a larger scale, evidence has accumulated that there are chromatin mechanisms that coordinate initiation from multiple origins in regions that can be as large as several 100 kb. These suggestions are based on studies of the sub-nuclear localization of replicons during S phase, on observations (using high-throughput methods) of large contiguous regions of the genome that seem to exhibit coordinated replication, and on the findings that rif1 knock-out or knock-down and non-coding RNAs can affect the replication times of large chromosomal domains [15]–[17]. Based on these results, the Gilbert lab has proposed the replication domain model which postulates the existence of distinct epigenetic entities (domains) with physical boundaries independent of replicon distribution. According to this model, epigenetic mechanisms would be able to control replication timing by influencing initiation of DNA replication from all the origins within these domains [18].

No consensus sequences have been demonstrated for all origins of replication in mammalian cells [19]. No consensus sequences were found in studies of short newly replicated nascent strands (NS) isolated from 1% of the genome in transformed cells [20] and on 0.4% of the genome in mouse embryonic stem cells [21]. Pioneering studies on about 2% of the mouse genome in several cell lines demonstrated an association between origins of replication and G-rich regions [10], [22]. In agreement, genome-wide studies by nascent strand DNA sequencing and bubble-seq demonstrated genome-wide association between CpG islands, transcription start sites, G4 quadruplexes and uncovered novel associations among origins, specific histone modifications and DNase I hypersensitive sites [23]–[25]. Although the primary sequence can clearly affect initiation capacity of individual replication origins [26]–[29], these findings raised questions regarding the relative contributions of genetic and epigenetic regulatory mechanisms in determining the locations, initiation frequencies and timing of initiation from replication origins.

The genome-wide timing profiles produced so far represented the average replication timing of the two alleles in a population of cells. In such profiles, regions that appear to replicate in mid-S phase can be interpreted either as regions in which the two alleles replicate at the same time in mid-S phase or as regions in which the two alleles replicate asynchronously or randomly. The size of asynchronously replicated domains and the mechanism of their establishment remain largely unknown. Three mechanisms are known to be associated with asynchronous replication of the two chromosome homologs: X chromosome inactivation, parental imprinting and mono-allelic expression [30], [31]. A recent genome-wide allele-specific study in human lymphoblastoid cell lines has shown that the inactive X chromosome, replicated in a less structured manner than the active X chromosome and early replicating regions [32]. Whether other mechanisms can lead to replication asynchrony is not known.

To characterize the extent of replication asynchrony in human cells and to begin to address the possible causes of asynchrony, we have developed an approach to measure allele-specific replication timing genome-wide. In addition, we have developed an approach to differentiate between genetic and epigenetic mechanisms based on the analysis of the phased genomes of a family quartet. We have used this method as well as two distinct origin mapping methods based on non-overlapping assumptions to provide a high resolution view of the regulation of replication in human primary cells.

## Results

### Generation of allele-specific profiles of timing of replication

**Figure 1:**
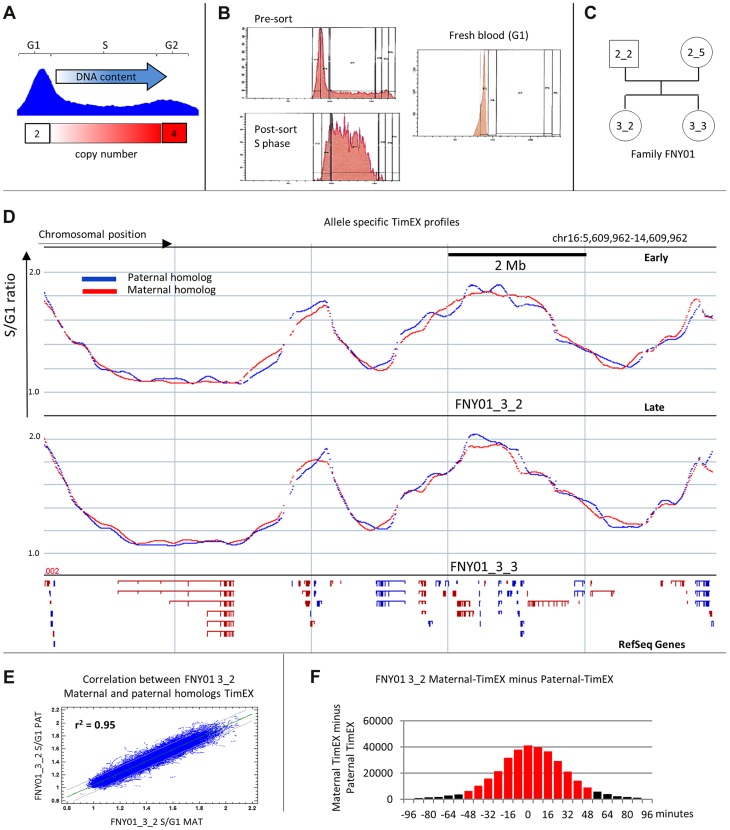
Allele specific timing profiles. A: Principle of the TimEX methods: DNA content from cells in the G1 and S phases of the cell cycle is compared. Cells in G1 contain 2 copies of each genomic region. By contrast, cells in S contain between 2 and 4 copies of each genomic regions and the number of copies in S is inversely proportional to replication timing. The S/G1 ratio can therefore be used as a surrogate for replication timing. B: Histograms representing the DNA content profiles of exponentially growing cultured basophilic erythroblasts (presort), of the same cells after sorting the S phase fraction (post sort) and of circulating white blood cells that are almost exclusively in the G1 phase of the cell cycle. Cells were labeled with Propidium iodide for 15 minutes. C: Pedigree of family FNY01. D. Plots illustrating the replication timing of the maternal and paternal homologs in a 10 Mb region on chr16 for FNY01 3_2 and 3_3. The data was generated by binning the read depth of heterozygous SNPs in 500 bp windows, applying a Gaussian smoothing filter (sigma = 100 kp) and calculating the S/G1 ratio for the maternal and the paternal homologs. The profiles are discontinuous because some reion of the genome did not contains any heterozygous SNPs. Replication of the homologs is tightly regulated. The location of Refseq genes is indicated below the timing profiles to give a sense of scale. E: Scatter-plots of the S/G1 ratios of the allele-depth of heterozygous SNPs in 5 kb windows for the maternal and paternal homologs. Coefficient of correlation between the two homolog is very high (r^2^ = 0.95). F: Histogram of the distribution of the difference between the S/G1 ratio of the maternal and paternal homologs. The two homologs replicate within 48 minutes of each other in about 88% of the genome. The black bars represent the asynchronous regions.

The TimEX method compares the amount of DNA present in the S and G1 phases of the cell cycle as a surrogate for the timing of replication. The approach was first implemented at a low resolution using micro-arrays [33] and then extended genome-wide to high-throughput sequencing [34] (Figure 1A & B). TimEX-seq measurements are very accurate because they do not require drug-induced cell synchronization, which can induce artifacts, or sorting of multiple S phase fractions, which can be difficult to calibrate. Genome-wide allele-specific analysis requires phased genome sequences which can be obtained by several methods including sequencing members of the same family [35]. To establish an experimental system that would allow allele-specific analysis in human primary cells, we recruited family FNY01, a large family with no known genetic diseases, completely sequenced the genomes of two sisters and their biological parents (Figure 1C) and phased all genetic variants using a combination of transmission, physical and population-based phasing methods [36]. The TimEX-seq method was then applied to human primary basophilic erythroblasts that were produced by *in vitro* differentiation of circulating peripheral blood hematopoietic stem and progenitor cells from two members of the family [37]. All of the timing experiments were therefore performed twice on two different (but related) individuals. Allele-specificity was achieved by only considering reads containing phased heterozygous SNPs that could be assigned to either the maternal or paternal chromosome homologs. For each individual tested, we obtained between 43 and 55 million reads that contained informative SNPs. Sequencing depth per called SNPs averaged between 23 and 28 for the S and G1 profiles (Table S1).

Allele-specific replication timing profiles were generated by calculating the number of reads from the maternal or paternal homologs obtained from cells in the S or G1 phase of the cell cycle in 500 bp genomic windows, smoothing the data with a Gaussian filter (sigma equal to 100 kb) and computing the S/G1 ratios of these smoothed allele-depth sums. Gaussian filters are similar to moving-average or Loess filters, but the averaged values are assigned weights that decrease following a Gaussian curve as a function of genomic distance.

SNP density in family FNY01 varies between 1 SNP every 500 bp and one SNP every 5 kb because of the haplotype structure of the human genome [36]. The resolution of the replication timing information obtained therefore depended on the SNP density and regions of very low SNP density resulted in gaps. We estimate that we obtained accurate TimEX values for about 85% of the genome.

The general shapes of the allele-specific profiles thus obtained were very similar to the shapes of the non-allele specific profiles that we and others previously generated (Figure 1D) [34], [38]. About 96% of the TimEX values were between 0.9 and 2.1 in good agreement with the theoretically expected range of 1 to 2. About 70% of the outliers were between 0.8 and 2.3 and fell mostly in regions of low SNP density.

The Pearson's correlation coefficient between the timing profiles of the maternal and paternal homologs was very high (r-squared = 0.95; Figure 1E). Histograms of the differences in TimEX values between the two alleles, in all 5 kb intervals genome-wide, had a mean equal to zero, but there was significant variation from normality at the extremes of the distribution reflecting the existence of regions in which the two alleles replicated asynchronously (Figure 1F and S1).

### The replication program of the two homologs is highly concordant

To assess the average level of asynchrony, we first calculated the difference in the timing profiles for the two individuals tested. Assuming an 8 hour S phase, the differences in timing of replication between the two alleles was less than 10% of the length of S phase in 88% of the genome. This suggests that the two homologs replicated within less than 48 minutes of each other. Therefore, at the resolution of this study, the vast majority of the genome replicated synchronously.

In the 12% of the genome that exhibited asynchronous replication, the timing differences did not generally exceed 30% of S phase length, corresponding to a maximum of 2–3 hours of replication delay between the two alleles. This was consistent with previous reports that showed that the time of replication of imprinted genes varies by only a few hours [39]. Larger differences in timing of replication likely exist between the active and inactive X chromosomes, but their detection would require the study of clonal cell populations.

### Large structural variants can affect replication timing

To characterize the mechanisms underlying replication asynchrony, we first attempted to determine if genetic differences between the two homologs could account for some of the replication asynchrony. There are four haploid genomes in a family quartet: two contributed by the mother and two by the father. The random assortment of chromosomes and the crossovers that occur during meiosis create recombined versions of these genomes in the children. Four inheritance states can be recognized [35]. When the two children inherit the same alleles from their mother and father, they are in the identical inheritance state; when they inherit different alleles, they are in the non-identical state; when they inherit the same allele from one parent but a different one for the other parent; they are in the haplo-identical maternal or paternal state. Each state represents about 25% of the genome of each child, the boundaries between the states being defined by the crossovers that occurred during meiosis in the parents.

**Figure 2:**
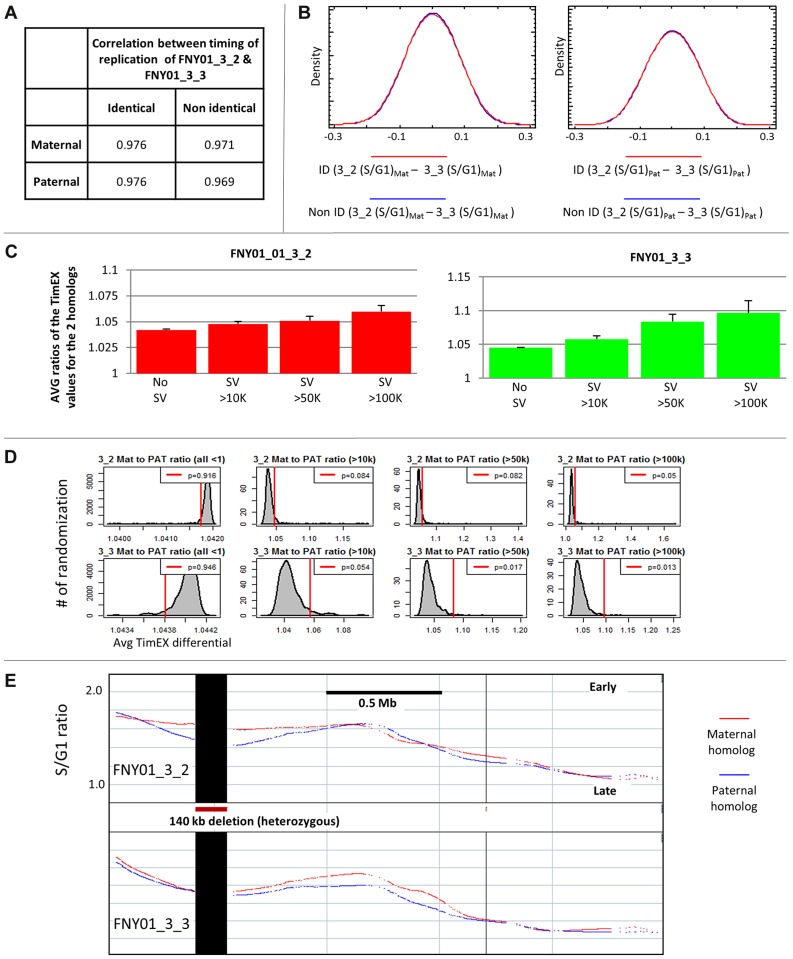
Presence of large structural variants can alter replication timing regionally. A: Comparison of the timing of replication in genetically identical and non-identical regions. Coefficients of correlation between the timing of replication of the two maternally inherited or the two paternally inherited chromosomes calculated in 500 bp windows in the identical and non-identical regions of the genome of FNY01 3_2 and 3_3. The timing of replication is very similar in the identical and non-identical regions. B: left panel: Density traces of the differences between the mean-centered TimEX values of the maternal homologs in individuals FNY01_3_2 and 3_3 in the identical (ID) and non-identical (Non ID) regions. Differences were calculated in 500 bp windows. The timing of replication of the maternally inherited chromosomes of FNY01 3_2 and 3_3 are as closely related to each other is the non-identical and identical regions. Right panel: same but for the paternal homologs. C: Histograms illustrating the mean (± S.E.M) of the avg timing of replication differential of the paternal and maternal homologs in 500 kb intervals containing either no SVs greater than 10 Kb (No SVs), or SVs larger than 10, 50 and 100 kb for individual FNY01_3_2. For each 500 kb interval, the timing differential was calculated as the average of the S/G1 ratio computed in 5 kb windows after inversion of the ratios smaller than one to capture the absolute value of the timing differential. Similar results were obtained using intervals of 1 Mb, using overlapping or non-overlapping intervals, and calculating the timing differential as log ratio, as a difference or as or as distance between the allelic TimEX values. Timing is highly synchronous in the 500 kb intervals containing no SVs and in the identical and non-identical regions. By contrast, regions containing SVs exhibit larger and larger differences between the replication times of the two homologs as the size of the SV increases. D: Statistical significance of the influence of SVs on timing of replication. Location of the SVs in the genome was randomized and the average timing differentials were calculated as above. Histograms summarize the average timing differential observed for 100,000 randomizations. P-values were calculated as the fraction of randomization that yielded average timing differential higher than the observed differential, Red lines represent the timing differential observed with the actual data. E: An asynchronously replicated region associated with a 140 kb deletion that is heterozygous in both FNY01 3_2 and 3_3.

To determine if genetic differences can affect timing of replication, we compared the timing of replication in the identical and non-identical portions of the genome. The coefficients of correlation between the maternally inherited chromosomes of FNY01 3_2 and 3_3 were very similar in the identical and non-identical regions despite the presence of more than a million SNPs and indels between the maternally inherited homologs in the non-identical regions (Figure 2A). Very similar results were found when the paternally inherited chromosomes were compared (Figure 2A) and when the calculated times of replication were compared in 500 kb windows rather than in 5 kb windows (Figures S2A). Likewise, the distributions of the differences in timing between the maternal homologs or between the paternal homologs in the identical and non-identical regions were almost indistinguishable (Figures 2B and S2B). We conclude from this comparison that the presence of SNPs and indels has no detectable on replication timing, at the resolution of this study. One likely explanation for this important result is that the timing of replication in any region is influenced by the combined initiations from many origins and therefore that differences in origin efficiency induced by genetic variations in the non-identical part of the genome cancel each other out and do not result in any detectable timing differences when large timing domains are considered. An alternative but less likely explanation is that there might be an evolutionary bias against mutations in origins of replication. Since the timing of replication can be determined by distal elements and epigenetic modifications, as well as by primary sequences [40], redundancy of regulatory elements might also contribute to the robustness of the regulation of the timing of replication. Additional experiments will be necessary to determine if SNPs and indels can affect initiation from individual origins on a genome scale.

Sequencing of family quartet FNY01 had revealed that there are about 1,500 structural variants (SVs) above 1 kb in size in the quartet, including about 100 variants larger than 10 kb, and 10 larger than 100 kb in each family member [36]. We took advantage of these naturally occurring variants to ask whether timing differences between the two homologs might be induced by large deletions and duplications. Examination of the ratios of the timing of replication of the homologs in overlapping 500 Mb intervals in regions containing heterozygous SVs revealed genomic regions in close proximity to SVs exhibited a higher prevalence of asynchrony than regions that were far from SVs (Figures 2C–E, S2C–D). This was observed in the two individuals tested. The effect was small in the case of 10 Kb variants but increased progressively as the size of the variants increased.

To determine if these observations were statistically significant, we performed randomization of the location of the SVs and calculated the ratios of the timing of replication of the homologs in overlapping 500 Mb intervals. This analysis demonstrated that the effect of SVs on timing of replication was statistically significant (Figure 2D). This suggests that some large SVs alter the timing of replication, maybe because deletions or insertions of multiple origins of replication have an additive effect. This result is consistent with distal regulation of DNA replication initiation events, since large structural mutations might affect chromatin modifications and spatial relationships with chromatin modifiers such as LCRs, enhancers, or insulators, which might in turn affect timing of more than one origin.

### Spatial and temporal resolution of the TimEX method

We then attempted to detect asynchronously replicated domains (ARDs), defined as genomic regions that exhibit asynchronous timing of replication of the same signs over large regions.

To automate and optimize the detection of these ARDs and more generally to assess the temporal and spatial resolution of the allele-specific TimEX-seq, we simulated ARDs in our data. These simulations were performed using a binomial random simulator. We first resampled the SNP-containing reads to create simulated maternal and paternal controls data tracks. Various amounts of reads were then added *in silico* to randomly selected genomic regions of one of these controls to simulate asynchronous regions ranging in size from 125 kb and 2 Mb. Simulated TimEX profiles were then generated by smoothing the data and calculating S/G1 ratios as described above for the actual data.

Once these simulated TimEX profiles had been created, we tested a variety of approaches to systematically detect the simulated ARDs. This resulted in the development of a highly efficient two-step method to detect ARDs. In the first step, the island finder of GenPlay is used to detect regions of differential timing in the smoothed profiles; in the second step, the statistical significance of the islands thus defined is assessed by performing chi-square tests on the sum of the reads in each island. Parameters to define the ARDs with the island finder (TimEX differences threshold, maximum gap size and minimum island size) were optimized by trial and error to maximize the rate of detection of the simulated islands.

These simulations revealed that the sensitivity of the allele-specific TimEX-seq approach is dependent on the size of the asynchronous regions and on the amplitude of the delay (Figure S3). A 10% excess in S phase DNA (corresponding to a timing asynchrony of 48 minutes), could be detected more than 90% of the time (with an FDR of 5%) for 2 Mb asynchronous regions. A similar detection rate for regions of 1 Mb and 500 Kb respectively required an excess of S-phase DNA of 15% (72 min. asynchrony) or 20% (96 min. asynchrony). Asynchronous regions smaller than 500,000 bp were difficult to detect. These simulations also revealed that the smoothing that we applied to the data created some distortion of the replication delay and size of the ARDs. The detected ARDs had a smaller timing delay and a larger size than the simulated asynchronous regions that we had introduced. These distortions were small for regions larger than 500,000 bp but quite large for smaller regions (Figure S3). Since ARDs smaller than 500,000 bp were difficult to detect and highly distorted by the smoothing process, we decided to limit our analysis to ARDs larger than 500,000 bp. Additional simulations revealed that detection of smaller size ARD would be possible using TimEX-seq but would require sequencing to a higher read-depth.

Because the average replication delays in the ARDs were under-estimated using the optimized detection parameters, we also used the Island finder in GenPlay to identify within the ARDs, core regions where the timing difference was highest. Timing delays in these cores are a better approximation of the true delay than the average delay in the entire ARD.

Having optimized the method to detect ARDs, we quantified the number of ARDs in individuals FNY01_3_2 and 3_3. There were 617 statistically significant ARDs in FNY01_3_2 and 611 in 3_3 (Table S2A–C). Two hundred sixty-two ARDs overlapped between the two individuals. The lack of overlap for some of the ARDs likely reflects technical issues (heterogeneity in the number of SNPs in each region caused by the haplotype structure of the genome) as well as genetic or epigenetic differences between the two individuals. The average timing delay in the ARDs and in the core ARD were, respectively, approximately 49 and 87 minutes. The maximum delays were around 2.5 hours in the ARDs and 3.5 hours for the core ARDs.

### Asynchronous replication domains are enriched in imprinted genes

**Figure 3:**
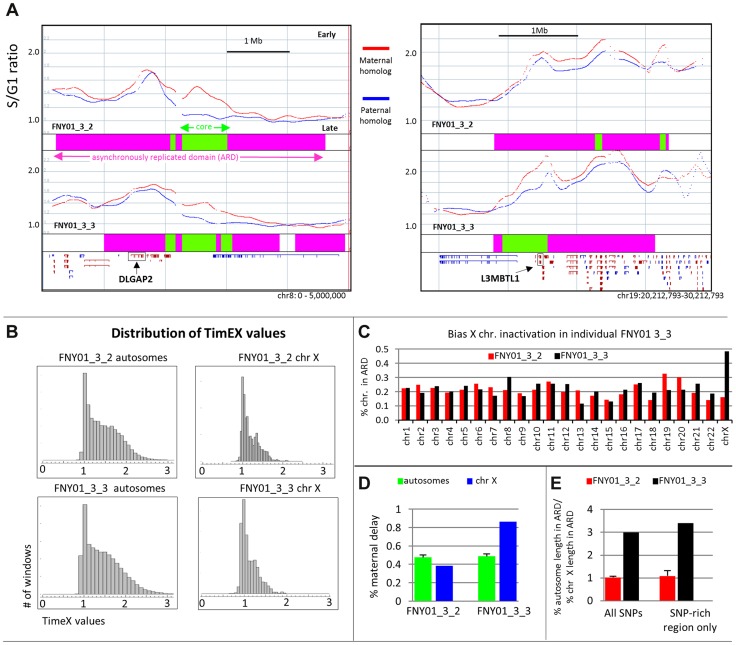
Large asynchronously replicated region contains imprinted genes. A: Plots illustrating two large asynchronously replicating regions containing two paternally imprinted genes DLGAP2 and L3MBTL1 in FNY01_3_2 (top) and 3_3 (bottom). The blue and red curves respectively represent the TimEX profiles of the paternal and maternal homologs (S/G1 copy number ratios after Gaussian smoothing (sigma = 100 Kb)). The pink boxes below the curve illustrate the Asynchronously Replicated Domains (ARD); the green boxes, the core ARD. Refseq genes are plotted to indicate the location of the imprinted genes. B: Histogram illustrating the distribution of the TimEX values for the autosomes and the X chromosomes. As expected, the distribution of the values for the autosomes is broader than for the sex chromosomes because one of the X chromosomes replicates uniformly late. C: Histogram illustrating the fraction of the length of each chromosome that is within an ARD region. This fraction varies between 0.1 and 0.3 for all chromosomes except for the X chromosome of individual FNY01_3_3 which contains ARD for almost half of its length. D: Histogram illustrating the fraction of ARD that exhibited a maternal delay for the autosome and for the sex chromosomes. Paternal and maternal delays are approximately equally distributed in all autosomes and in the X chromosome of FNY01_3_2. By contrast almost 90% of the ARD in chromosome X of FNY01_3_3 exhibit a maternal delay. E: Histogram illustrating the ratios of the percentages of the length of the autosome that is asynchronously replicated to the percentage of the X chromosome that is asynchronously replicated in FNY01_3_2 and 3_3 considering all the SNPs or only the SNPs rich regions. The imbalance in the percent X chromosome that is inactivated of FNY01_3_3 can be detected even when only the SNP-rich regions are taken into consideration. Together Figure 3B to 3E strongly suggest that X inactivation in the erythroid lineage of individual FNY01_3_3 is biased toward the maternal chromosome which is inactivated more often than the paternal chromosome.

To determine if asynchronous timing of DNA replication was associated with imprinting, we analyzed the distribution of imprinted genes in the ARDs. To increase read-depth we created a combined track in which the reads of both individuals were summed together. One hundred and one known or predicted imprinted genes showed evidence of asynchronous replication. Sixty-three known or predicted imprinted genes were within ARDs in FNY01 3_2, forty-three in FNY01 3_3 and sixty-five in the combined track (Figure 3A, Table 1 and S3). Fifty-six genes were in imprinted domains in both individuals or in one individual and in the combined track. In addition, a number of genes, most notably part of the H19 cluster, exhibited evidence of asynchrony (delays of over 45 minutes over large regions) but the domains did not reach statistical significance because of low SNP density.

**Table 1:** Most significant asynchronously replicated ARD associated with imprinted genes.

FNY01_3_2										
ARD Name	chr	start	stop	length	p-values	q-values	Imprinted Gene	coverage	ARD delay (minutes)	core delay (minutes)
chr19.7	chr19	20,858,999	23,376,499	2,517,500	7.14E-45	3.15E-41	ZNF738	0.42	−85.06	−135.90
chr8.0	chr8	220,999	4,633,499	4,412,500	1.99E-40	4.39E-37	DLGAP2	0.45	54.35	93.09
chr6.41	chr6	105,669,499	107,304,999	1,635,500	2.73E-15	2.41E-12	LIN28B	0.28	−75.76	−118.59
chr7.15	chr7	26,896,499	27,401,499	505,000	7.55E-11	2.56E-08	HOXA2	0.26	−117.47	−160.48
chr10.45	chr10	121,596,999	125,299,999	3,703,000	1.63E-09	4.22E-07	INPPF5	0.29	40.02	89.20
chr20.21	chr20	60,497,999	62,269,999	1,772,000	7.51E-08	1.38E-05	MRGBP	0.24	−91.69	−120.92
chr9.2	chr9	2,409,499	4,119,999	1,710,500	1.33E-07	2.35E-05	GLIS3	0.30	−40.84	−40.84
chr19.10	chr19	33,587,499	37,609,499	4,022,000	3.48E-07	4.80E-05	CHST8	0.30	42.10	92.26
chr17.3	chr17	7,727,999	8,491,999	764,000	2.27E-06	2.33E-04	TMEM88	0.27	104.49	124.08
chr20.12	chr20	41,494,499	44,024,499	2,530,000	4.69E-06	3.76E-04	L3MBTL1	0.24	57.42	85.41

FNY01_3_3										
ARD Name	chr	start	stop	length	p-values	q-values	Imprinted Gene	coverage	ARD delay (minutes)	core delay (minutes)
chr19.32	chr19	19,967,999	23,373,499	3,405,500	1.37E-54	5.87E-51	ZNF738	0.37	−87.0	−77.1
chr8.2	chr8	1,008,999	3,885,499	2,876,500	1.32E-36	2.84E-33	DLGAP2	0.43	60.4	93.8
chr3.215	chr3	146,925,999	148,541,999	1,616,000	2.77E-10	6.53E-08	ZIC1	0.21	59.5	87.3
chr20.62	chr20	41,479,499	43,830,499	2,351,000	6.45E-10	1.38E-07	L3MBTL1	0.22	78.0	127.2
chr15.5	chr15	24,870,999	25,861,499	990,500	6.01E-07	5.74E-05	NPAP1	0.20	64.9	82.6
chr19.67	chr19	44,655,499	45,682,499	1,027,000	3.98E-06	0.0002	ZNF229	0.30	76.0	92.4
chr1.65	chr1	39,749,499	42,207,999	2,458,500	1E-05	0.0005	BMP8B	0.24	−56.3	−95.5
chr7.152	chr7	93,861,499	94,433,999	572,500	3.99E-05	0.0018	SGCE	0.23	64.2	78.8
chr6.169	chr6	104,288,999	105,477,999	1,189,000	0.0001	0.0037	LIN28B	0.33	−37.1	−37.1
chr8.199	chr8	139,563,499	140,884,999	1,321,500	0.0001	0.00424	KCNK9	0.24	−44.6	−96.8

The most statistically significant ARD and the most asynchronous ARD were enriched in imprinted genes. When the ARDs were ranked by p-values, respectively eight and seven of the 40 most significant asynchronously replicating domains in individual FNY01_3_2 and 3_3 contained (or were within 100 kb) of an imprinted gene. Randomization experiments revealed that this enrichment was highly statistically significant (p-values = 7.90E-4 for FNY01_3_2 and 1.68E-4 for FNY01_3_3).

Computation of the average length of ARDs that were associated or not associated with imprinted genes revealed that the former category of ARD genes was about 1.5-fold larger (Table 2). These differences were highly statistically significant (t-test p-values <10^−5^).

**Table 2:** Comparison of ARD containing and not containing imprinted genes.

	ARD containing imprinted genes	ARD not-containing imprinted genes	p-values (t-test)
**FNY01_3_2**			
**Avg ARD length**	1.4.±0.14 Mb	1.0±0.02 Mb	**2.34E-06**
**Avg ARD delay**	54.5±3.4 min.	48.5±0.8 min.	**5.00E-02**
**Avg core ARD delay**	81.3±6.9 min.	69.-±1.8 min.	9.00E-02
**FNY01_3_3**			
**Avg ARD length**	1.6±0.20 Mb	1.1±0.26 Mb	**1.56E-04**
**Avg ARD delay**	54.2±4.2 min.	48.1±0.9 min.	1.10E-01
**Avg core ARD delay**	85.6±8.5 min.	65.8±1.6 min.	**7.00E-03**

The average delays for the ARD and for the core ARD was about 15–30% higher for ARDs that contain imprinted genes when compared to those that did not contain imprinted genes. These differences reached statistical significance for both individuals (Table 2). We conclude from this analysis that imprinted genes often localize in the longest, most asynchronous ARD. However, many long highly asynchronous ARD did not contain imprinted genes.

### X chromosome inactivation

The erythroblasts profiled in this study are the progeny of thousands of primary stem and progenitor cells seeded in our 2-week erythroid culture system. Because inactivation in placental mammals is random, the TimEX profiles of the paternal and maternal homologs both represent the average timing of replication of the Xa and Xi chromosomes. We therefore did not expect to distinguish between the active (Xa) and inactive (Xi) X chromosomes unless X inactivation was imbalanced. Imbalanced X chromosome inactivation can occur stochastically or because of selection [41], [42]. Skewed inactivation have been described in the erythroid lineage, for instance in the case of heterozygous G6PD deficiency [43].

Since the Xi chromosome replicates generally later than the Xa chromosome, which can replicate throughout S phase [44], [45], the range of the S/G1 values for the Xi chromosomes is expected to be narrower than that of the autosomes. To verify this prediction in our experimental system, we plotted the histograms of the S/G1 ratios for the autosomes and for the X chromosomes (Figure 3B). As expected, the S/G1 ratios varied from about 0.9 to 2.2 for the autosomes and only between 0.9 and 1.5 for the X chromosomes, suggesting that one of the X chromosomes replicated relatively uniformly late in both individuals, consistent with recent whole-genome observations in adult lymphoblastoid cell lines [32].

Further analysis revealed a chromosome X specific difference between individuals FNY01_3_2 and 3_3, who are both female. The ARDs on the X chromosomes of FNY01_3_2 were similar to those of the autosomes: They represented 10–30% of each chromosome and there was approximately the same number of ARDs exhibiting maternal or paternal delays on each chromosome (Figure 3C and 3D). By contrast, almost 50% of the length of chromosome X of individual FNY01_3_3 replicated asynchronously and almost 90% of the ARDs on chromosomes X of FNY01_3_3 exhibited a maternal delay. Together, these observations strongly suggest that X-chromosome inactivation in the basophilic erythroblasts of FNY01-3_3 that we tested was not random but was skewed toward inactivation of the maternal chromosome. To determine if this skew was due to the lower SNP density on the X chromosomes relative to the autosomes, we masked the SNP-poor regions and repeated the analysis. As shown in Figure 3E, the same pattern was observed, suggesting that the observation is not a technical artifact of the TimEX method.

The cellular and molecular mechanisms responsible for this inactivation bias are unclear. The bias might have been acquired in early development, in the hematopoietic stem cells or in culture during erythroid differentiation, maybe because FNY01_3_3 is heterozygous for an X-linked gene such as G6P-D that is selected against in the erythroid lineage. In any case, these observations suggest that allele-specific TimEX will prove useful to assess of inactivation of the X chromosomes in human primary cells.

### High-resolution TimEX reveals ripples within the timing domains

**Figure 4:**
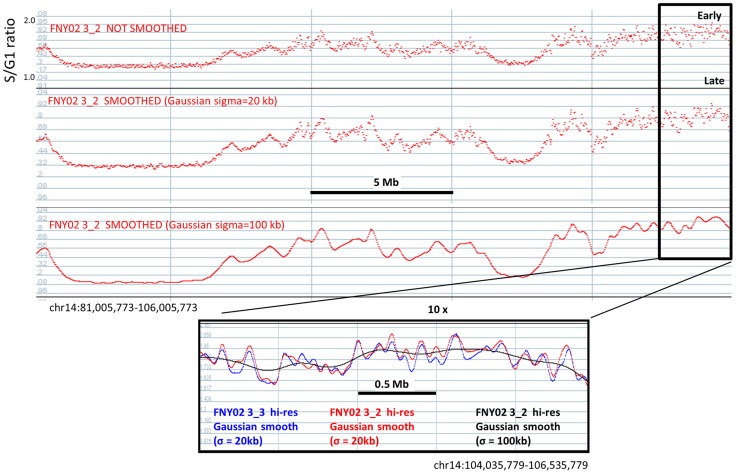
Hi-resolution TimEX-seq analysis reveals the fine structure of the timing domains. 25-resolution TimEX profiles. Top panel: S/G1 ratios calculated every 1 kb are plotted. At this read depth, the shape of the timing domains can be distinguished without any smoothing. Middle and bottom panel: same data after Gaussian smoothing using sigma = 20 Kb or 100 kb. Smoothing at 100 kb, which was necessary to obtain reproducible profiles after read-depth at a low read depth or with micro-array based methods, reveals the major timing domains but smooth the details out. Inset: red curve: TimEX profile after 20 Kb Gaussian smoothing for individual FNY01 3_2; black curve: same data after 100 kb Gaussian smoothing; blue curve: TimEX profile for individual FNY01 3_3 after 20 kb Gaussian smoothing. Note the reproducibility sub-peaks or ripples in the profiles of FNY01 3_2 and 3_3 and how the 100 kb processing masks the details.

Production of allele-specific timing profiles required a sequencing depth of more than 6.10^11^ bases (25–30x coverage per individual) because only about one read in ten contains an informative SNP. By considering all the reads produced by the experiment, we also constructed high-resolution timing maps that were not allele-specific but that were more precise than any map previously produced. The resolution of these profiles was so high that very little smoothing was required (Figure 4). Calculating the S/G1 ratios in 1 kb windows and smoothing lightly with a sigma of 20 kb, instead of 5 kb windows and a sigma of 100 kb [34], revealed reproducible sub-peaks, or ripples within the megabase-size timing domains that have been well-characterized by several labs (Figure 4) [38], [46]–[49]. By analogy with Raghuraman et al. who have shown in yeast that maxima of timing of replication profiles correspond to the location of origin of replication [50], we hypothesized that these ripples, which had not been previously reported, might co-localize with zones of origins of replication or particularly dominant origins of replication. To test that hypothesis, we generated maps of replication origins, validated the data by comparison with previously published results, and compared TimEX and origin of replication profiles.

### Profiling origins of replication in basophilic erythroblasts using two independent methods

**Figure 5:**
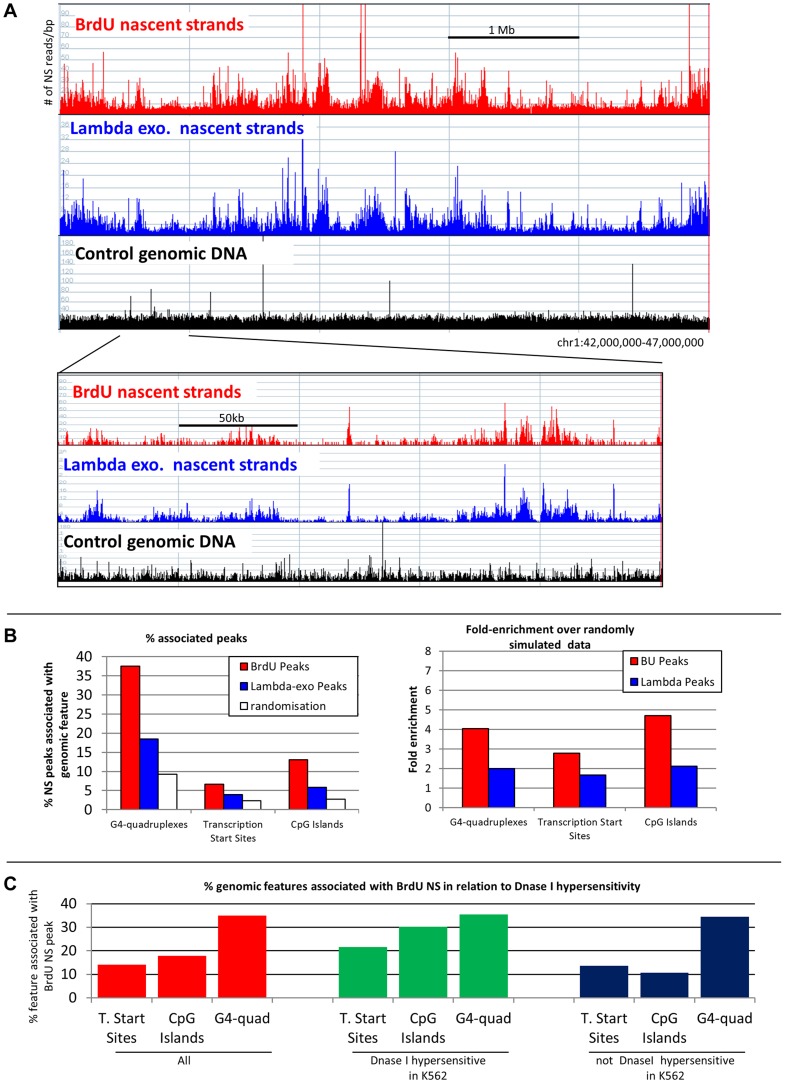
NS profiles. A: Comparison of NS prepared using the lambda-exonuclease and BrdU methods. Profiles of newly replicated NS DNA in a 5 million bases pair region on chr 1 (250,000 for lower panel). Red bars: NS 0.5 to 1 kb in length were isolated by immuno-precipitation of newly replicated DNA labeled with BrdU. Blue bars: NS prepared using the lambda exo-nuclease method. Black bars: Sheared genomic DNA (mapability control) B: NS peaks are associated with 3 genomic features: left panel: histograms illustrating the association of G-quadruplexes, transcription start sites and CpG islands with nascent strand peaks generated by the BrdU (red bars) or by the lambda exonuclease (blue bars) methods. Bootstrap (white bars) = average association with randomly simulated data (100–1000 iteration gave the same results). Y-axis = percent BrdU NS peaks associated with a feature (100× # of NS peaks associated with feature/# of NS peaks). Right panel: enrichment over bootstrap: Y axis = (% associated peaks/% associated random peaks). C: Chromatin accessibility favors the formation of origins of replication at G-quadruplexes, transcription start sites and CpG islands: Red bars represent the percent of G-quadruplexes, transcription start sites and CpG islands that are associated with a BrdU NS peaks. Green and blue bars respectively represent the percent of G-quadruplexes, transcription start sites and CpG islands that are (green) or are not (blue) located within a DNase I hypersensitive site and that are associated with a BrdU NS peaks. DNase I hypersensitivity data is from K562 cells. Y-axis = % features associated with BrdU NS peaks.

To obtain maps of replication origins, we cultured basophilic erythroblasts from FNY01_2_2, the father of FNY01_3_2 and 3_3, and generated genome-wide profiles of nascent, newly replicated DNA strands. Because of the reported difficulties involved in reproducing NS profiles between labs, two methods were used. Replication initiation analyses were performed with two technical replicates, using two methods relying on non-overlapping assumptions to isolate nascent DNA strands. Nascent strands were first purified using the classic method which relies on sequencing DNA fragments of small size that contain RNA primers that rendered them resistant to lambda exonuclease digestion [23]. To control for potential biases introduced by the use of lambda exonuclease, we also mapped NS with a method that does not rely on this enzyme. Briefly, the cells were pulse-labeled for 30 minutes with bromo-deoxy-Uridine (BrdU), and short, newly synthesized DNA strands were immuno-precipitated with anti-BrdU antibodies, yielding a population of NS that were also subject to massively parallel sequencing [40], [51]–[53]. Results from both methods were in good agreement. The genome-wide coefficient of correlation between the profiles obtained with both methods was 0.7 and reached 0.85 for several chromosomes (Figure 5A).

### Association of origins with G-quadruplexes, transcription start sites and CpG islands

Besnard et al. have reported a two-fold enrichment over randomly simulated data in the number of 4 kb fragments containing lambda exonuclease resistant NS peaks associated with G-quadruplexes and a three-fold enrichment for peaks associated with CpG islands and with transcription sites [24]. Similarly, Messner et al., using bubble-seq sequencing, have detected enrichments for these three genomic features in origin-containing 3 to 4 kb Eco RI fragments analyzed by Bubble-Seq, albeit of lower magnitude [25].

To compare the BrdU method to the lambda exonuclease method, we called the peaks observed with both methods using the MACS 1.42 software [54]. The false discovery rate (FDR) was set to 0.1%. The BrdU and lambda-exonuclease based methods yielded 249,000 and 290,000 peaks, respectively. Close examination of the peaks revealed that many of the statistically significant peaks were minor peaks that were barely above background. To simplify the comparison, we increased the size of all peaks to 400 base pairs and only considered the top 100,000 peaks for further analysis.

37.5, 6.6 and 13.1 percent of the BrdU NS peaks were, respectively, associated with G-quadruplexes, transcription start sites and CpG islands (Figure 5B). Enrichments over randomly simulated data were, respectively, 4.0, 2.8 and 4.7-fold (Figure 5B). About 46% of all peaks were associated with at least one of these three features (Figure S4A). Lambda-exonuclease peaks were associated with the same three features but the enrichment levels were lower (Figure 5B). Most of the prototypical origins [26], [55]–[58] that have been studied in details contained one or more of these genomic features (Figure S4B).

### DNase I hyper-sensitivity is associated with a subset of replication origins

Importantly, only 14.2, 17.8, and 35.1% of all G-quadruplexes, transcription start sites and CpG islands were, respectively, associated with BrdU NS peaks (Figure 5C). Only about one G-quadruplex in seven was associated with a BrdU NS peak. Overall, these data are consistent with the notion that subsets of G-quadruplexes, transcription start sites and CpG islands that are associated with origins of replication might have special characteristics.

To test whether chromatin accessibility contributed to origin formation, we next determined the percentages of G-quadruplexes, transcription start sites and CpG islands that were associated with BrdU NS peaks produced in our study with the DNase I hypersensitivity generated by the ENCODE consortium in either K562 cells or in fetal liver basophilic erythroblasts. These two cell types were selected because they are very similar to the adult basophilic erythroblasts that we studied here. This analysis revealed that the proportions of G-quadruplexes and transcription start sites associated with both BrdU NS and K562 DNase I hypersensitivity peaks were respectively multiplied by 1.52 and 1.69 (Figure 5C). Interestingly, chromatin accessibility barely increased the proportion of CpG islands associated with BrdU NS peaks. We therefore conclude that chromatin accessibility facilitates origin formation near G-quadruplexes and transcription start sites. Similar results were obtained when DNase I hypersensitivity data obtained from fetal liver basophilic erythroblasts was used (Figure S4C). It is noteworthy that while DNase I hypersensitivity was strongly associated with origin formation, the majority of origins of replication are not DNase I hypersensitive (Figure S4D). We conclude that chromatin accessibility favors but is not necessary for origin formation.

### Timing ripples co-localize with clusters of replication origins

**Figure 6:**
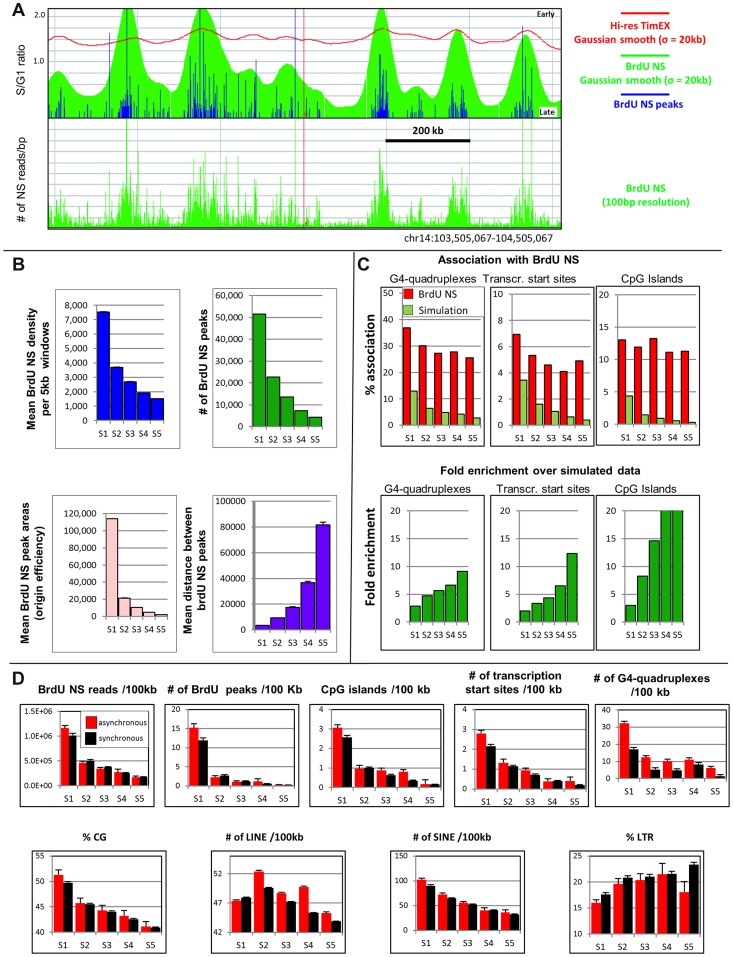
Comparison of TimEX and BrdU nascent strands profiles. A: Correlation of TimEX-seq and NS profiles: Top panel: Red curve TimEX-seq profile in a 1 Mb region of chr. 14 (Avg of the S/G1 values of FNY01 3_2 and 3_3). Green histogram: BrdU nascent strand smooth with Gaussian filter (σ = 20 kb). Blue histograms: Top 100,000 called nascent strand peaks. Bottom panel: BrdU NS profile in same region at 100 bp resolution. The timing peaks co-localize with clusters of BrdU NS. B: Top left panel: mean NS density per 5 kb window during S phase progression (S1 to S5 fractions were computed on the combined TimEX profiles of individuals FNY01 3_2 and 3_3). Top right panel: number of NS peaks in fractions S1 to S5. Bottom left panel: average NS peak area in fractions S1 to S5. Bottom right panel: mean distance between NS peaks. C: Association of BrdU NS with G-quadruplexes, transcription starts sites and CpG Islands. Association between BrdU NS peaks and these three genomic features occurs throughout S phase, even in S5 phase where very little transcription occurs. Top panels: Y-axis = % BrdU NS peaks associated with G-quadruplexes, transcription starts sites or CpG Islands. Bootstrap = average of the results of 100 random simulations. Bottom panels: Y-axis = fold-enrichment over average results of 100 random simulations. D: Analysis of asynchronous regions. The combined data from FNY01_3_2 and 3_3 analysis are illustrated. Similar results were observed when the data from FNY01_3_2 and 3_3 were analyzed separately. The top histograms from left to right respectively represent the density of BrdU NS reads/kb; the number of BrdU peaks/Mb; the number of CpG islands/100 kb, the number of transcription start sites/100 kb and the number of G-quadruplexes/100 kb as a function of replication time. The bottom histograms from left to right respectively represent the percent CG, LINE, SINE and LTR as a function of replication time. CG content was calculated in 10 kb interval using a 100 kb moving average window. The percent LINE, SINE and LTR was calculated as 100×(number of repeat base)/kb. The error bars represent the standard deviation of the mean.

We next analyzed the relationship between origins of replication and the ripples within the large timing domains that we detected in the high-resolution TimEX profiles. As illustrated in Figure 6A, visual examination suggested that the ripples in the timing profiles co-localized with clusters of origins of replication rather than with unique origins. This was not surprising since origins in mammalian cells often occur in zones of replication initiation [59]. Comparison of the high-resolution TimEX-seq with the BrdU NS data windowed at 3 kb revealed a high statistical correlation (r = 0.40, p<10E-5) between the earlier replicating regions of the high-resolution timing map and the NS. Smoothing the BrdU NS track to 20 Kb, to match the resolution of the high-resolution timing data, raised the coefficient of correlation to 0.54 (Table S4).

To measure overlap between the timing ripples and the BrdU NS clusters, we called the summits of the ripples and of the clusters of NS in the smoothed BrdU NS data (Figure S5). We also computationally divided the TimEX profiles into fractions S1 to S5 each containing 20% of the genome, with S1 as the earliest replicating fraction. Analysis of this data revealed that most of the timing ripples and NS clusters were in the S1 and S2 fractions, and that 68% of the ripples in S1 overlapped with the NS clusters (Figure S5). Since the overlap expected by chance was only 12%, we conclude that the reproducible ripples detected in the high-resolution TimEX profiles reflect the activity of clusters of highly active early-replicating origins of replication.

### Replication initiation occurs at a higher frequency in early replicating regions

To characterize origin distribution in relation to timing of replication, we computed the density of NS reads per 5 kb intervals, the number of NS peaks, and the distance between called peaks. We also computed the mean area of the NS peaks which is an approximation of origin efficiency. This revealed dramatic differences in the distribution of the NS peaks between the five S phase fractions with almost exponential decreases in the average density of NS per genomic intervals, in the number of the NS peaks, and in origin efficiency (Figure 6B). Conversely, the distance between NS peaks increased as S phase progresses. There were 12 times more NS peaks that were 38 times larger in S1 than in S5. Finally, the median inter-peak distance was about 500 bp in the S1 fraction and about 14,000 bp in the S5 fraction. Therefore, origins of replication are much more numerous and much more efficient in early than in late S phase. Generally, these results were in excellent agreement with the recent results of [22], [24]. However, since origin usage at the single molecule level is heterogeneous, the average inter-origin distances that we report here are likely smaller than the distances that could be measured on individual molecules.

The dramatic changes in origin number and efficiency according to replication time raised the question of whether early and late origins have similar association with G-quadruplexes, CpG Islands and transcription start sites. Analysis revealed that the associations between BrdU NS peaks and G-quadruplexes, transcription start sites, and CpG Islands were similar regardless of replication time (Figure 6C).

### Characterization of the asynchronously replicated regions

Besnard et al. have shown that regions that display tissue-specific timing of replication have fewer origins of replication than regions that have generally invariant timing in multiple cell types, suggesting that variation in timing of replication might occur preferentially in origin poor regions. [24]. We therefore hypothesized that a paucity of origins of replication might also be a cause of asynchrony. To test this hypothesis, we computed the number of BrdU peaks and the density of BrdU reads/kb for the synchronous and asynchronous parts of the genome using the data from either FNY01_3_2, FNY01_3_3 or the combined TimEX data for FNY01_3_2 and 3_3. The asynchronous part of the genome being defined as all regions located within an ARD.

As expected from the analysis of the high-resolution timing profiles depicted in Figure 6B, analysis of allele-specific timing profiles revealed that the average number of BrdU NS peaks and the average density of reads/peak was much higher in early than in late replicating regions in the synchronous part of the genome. The asynchronous regions exhibited a similar pattern. But the number of origins and the NS read density were slightly higher in the asynchronous region (Figure 6D). Therefore, asynchronously replicating regions were not associated with low initiation frequency, but, on the contrary, tended to contain more origins of replication than their synchronous counterparts. These conclusions apply to the population level and might need to be tested at the single molecule level.

We then asked whether asynchronous regions contained more G-quadruplexes, transcription start sites, and CpG islands than the synchronous regions. In accordance with the results of Figure 5, the synchronous regions were on average richer in all three genomic features in early compared to late regions. Again, the asynchronous regions followed the same pattern but contained higher counts of all three genomic features (Figure 6D). Finally, we asked whether asynchronous regions had a different CG and repeat content than the synchronous regions. Synchronous and asynchronous regions had very similar CG and repeat contents: CG and SINE contents were much higher in early compared to late regions, while LINE and LTR followed the inverse pattern (Figure 6D). These variations in CG and repeat content were generally in accordance with previously published results [60]. Very similar results were observed for both individuals tested and when the combined results were analyzed.

**Figure 7:**
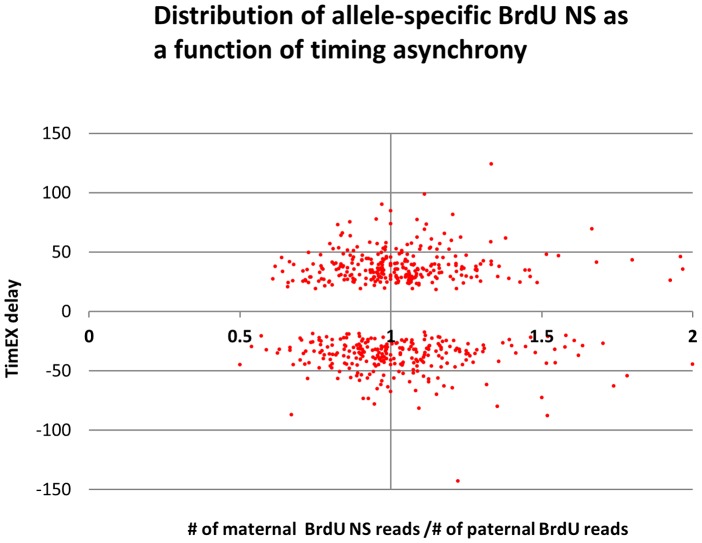
Distribution of allele-specific BrdU NS as a function of timing asynchrony. X-axis = # of maternal NS reads/# of paternal NS reads. Y axis = TimEX delay S/G1 ratio of maternal chromosome - S/G1 ratio of paternal chromosome. Each point represents one of the ARD regions observed in the combined FNy01_3_2 and 3_3 data. Similar results when the data from FNY01_3_2 or 3_3 were analyzed separately or when only the top 100 significant ARD or the imprinted ARD were considered. There is no correlation between maternal and paternal TimEX differential and the density of maternal or paternal BrdU NS.

To determine whether the NSs were differentially distributed on the two homologs in the asynchronously replicated regions, we called and phased the SNPs on the BrdU NS data files, and we generated allele-specific maps of origins of replication for the maternal and paternal homologs. To assess the average NS density over large regions, we plotted the ratio of the number of NS reads in each of the ARDs as a function of the TimEX delays observed in FNY01_3_2 and 3_3. Results demonstrated that the average NS read density was very similar on the two homologs (Figure 7). There was no correlation between the sign of the delays between the maternal and paternal homologs TimEX-seq signals and the ratios of the maternal and paternal BrdU NS. We conclude that timing asynchrony is not associated with a generalized change in origin efficiency on the two homologs.

## Discussion

Our results show that the timing of replication is very robustly coordinated in most of the genome. The non–allele specific high resolution profiles of FNY02_3_2 and 3_3 were almost identical. We show for the first time that in about 88% of normal somatic cells, the two chromosome homologs replicate at the same time during S-phase. This conclusion applies to the portion of the genome that we could access with our methodology and can likely be extrapolated to the entire euchromatic genome. Replication of constitutive heterochromatin cannot be assessed with current sequencing technologies.

Comparison of the identical and non-identical genomic regions of the two siblings that we analyzed revealed that, at the resolution of this study (about 400 kb), differences in the sequences of individual origins do not generally have a detectable effect at the level of the timing domains. Similarly, recent observations suggest that in human lymphoblastoid cells, replication profiles among autosomes (and the active X chromosome) were very similar when different individuals were compared [32]. Taken together, these observations imply either that the primary sequence of replication origins is only a minor determinant of replication initiation efficiency, or that alterations in the firing initiation rate of an origin can be compensated for by nearby origins and therefore do not affect the overall timing of replication in large replication domains.

In contrast, we observed that large SVs were associated with timing asynchrony that could be detected in genomic segments up to one Mb suggesting that the replication timing domains can be altered by large genetic differences that affect multiple origins but not by change in the efficiency of single origins. These results support the idea that timing can be regulated by mechanisms acting at the level of entire domains since it demonstrates that replication time is influenced by sequences that are hundreds of kilobases away.

To quantify asynchronous replication further, we developed a quantitative method to identify statistically significance ARDs and optimized the detection of these ARDs using simulated data.

This approach revealed that there was about 600 ARDs larger than 500,000 bp in the genome of the two individuals tested. ARDs could reach several megabases in size. About half of the ARDs overlapped in the 2 individuals tested. About 20% of the genome was located in ARDs. This number is larger than the 12% asynchrony discussed above because our method to call the ARDs tends to overestimate their sizes and because the statistically significantly asynchronous domain encompassed smaller regions that do not appear asynchronous when analyzed in isolation.

About 10% of the ARDs contained at least one known or predicted imprinted gene. ARDs that contain imprinted genes were longer and tended to be more highly statistically significant than ARDs that did not contain imprinted genes, suggesting that parental imprinted generates some of the most asynchronous regions in the genome. However, the vast majority of ARDs was not associated with large SVs or with imprinted genes. The mechanism of formation of these ARDs is unclear.

The presence of these 600 large asynchronous regions is a strong argument in favor of the existence of regulatory mechanisms that act at the domain level to control the times of initiation, but are difficult to interpret in the context of a strict independent origin model because hundreds of replication origins have to be coordinated to generate megabase-size domains of asynchrony.

Analyses of initiation profiles revealed that mapping newly replicated nascent strand DNA with the BrdU approach yields distinct patterns that are correlated to those obtained using lambda exonuclease analyses. Both approaches confirmed and extended previous observations that origins of replication are enriched near G-quadruplexes, CpG islands and transcriptional start sites (TSS). Almost 50% of narrowly defined DNA segments that contain origins of replication detected by the BrdU method were near at least one of these three genomic features.

These associations might be caused by the fact that G-quadruplexes, CpG islands and TSSs share sequence or chromatin characteristics that are necessary for origin formation. Alternatively, they might reflect an evolutionary selective advantage that stabilizes distinct genomic features (e.g. prevent replicative destabilization of G-quadruplexes [61]) when placed near replication initiation sites. The association of replication events with regulatory chromatin features could also reflect distinct origin types that would each be associated with particular genomic features and coordinate DNA replication with transcription and chromatin condensation [23].

The association between origins of replication and G-quadruplexes, CpG islands, and TSSs was higher in regions of open chromatin, as assessed by DNAse I hypersensitivity, suggesting that the presence of nucleosomes can decrease the probability of origin firing. However, the majority of origins were not located in Dnase I hypersensitive regions suggesting that the replication machinery can overcome these barriers.

Importantly, both the BrdU and lambda exonuclease approach revealed that replication initiation takes place near promoters and CpG Islands more often than expected by chance throughout S phase, including during late S phase in regions where no transcription occurs. This implies that the replication machinery recognizes promoters and CpG islands even when they are in a closed chromatin. The association between origins, promoters and CpG islands is therefore not dependent on the transcriptional function of these regions. Whether the replication machinery directly recognize the primary DNA sequence of these regions or epigenetic marks that retain the memory of previous transcription events remains to be determined. Methylation of histone H3 on lysine 79, which associates with some replication origins might be part of this process [62].

In any case, these data show that the primary DNA sequence and local chromatin structure at the nucleosome levels are critical determinants of where each origin of replication can be located. Therefore, the putative domain-wide epigenetic mechanisms that regulate initiation of replication likely act by modulating the efficiency of origins that are located in DNA regions specified in large part by the primary DNA structure.

Analysis of the frequency and efficiency of origins of replication in the ARDs revealed that there were more origins that were in average more efficient in asynchronous than in synchronous regions. Therefore ARDs do not occur preferentially in regions of low origin density.

Analysis of the high resolution timing profiles revealed a novel correlation between ripples in the timing of replication profiles and clusters of peaks in the nascent strand profiles. This important observation provides an orthogonal validation for the TimEX-seq and the BrdU and nascent strand approaches, and a novel method to detect initiation zones in timing profiles.

The large majority of the nascent strands that we detected were in early replicating genomic regions. The number of reads in NS peaks in late S phase was less than one percent that in early S phase because the peaks were larger and spaced closer together in early regions. Although our measurement of average levels in a population of cells mask the heterogeneity that can be observed at the single molecule level, the much larger quantity of NS that we detected in early replicating regions requires an explanation since DNA replicates only once per cell cycle.

One hypothesis is that late replicating regions are in part replicated passively from initiation events that occurred earlier in the cell cycle. This likely contributes to the excess NS in early S phase but the magnitude of this excess and the size of the late replication regions which are often too far from early origins to be replicated passively, suggest that other factors might also be at play. An additional hypothesis is that transcription competes with DNA replication in early S phase since both of these process relies on very large protein complexes that cannot access the DNA strand at the same time. This latter hypothesis is attractive because it also provides a potential explanation for the apparent stochastic nature of initiation within replication initiation zones observed at the single molecule level, since interactions between two independent processes is a well-established deterministic explanation for apparently stochastic events.

Interference with transcription and its associated complex chromatin structure could lead to increased origin activity and higher production of NS in early regions by slowing down the replication, thus providing the opportunity for nearby origins to fire rather than being replicated passively. In addition, a slower rate of progression of the replication fork would increase the half-life of short nascent strands and therefore increase their detection by both the lambda exonuclease and the BrdU approaches. The larger number of origins of replication and the higher efficiency of firing that we observed in early versus late S phase might therefore be due to both increased production and increased detection of the nascent strand in early S phase.

A higher number of initiation events in early S phase as compared to late S phase would lead to a smaller replicon size in early S. Several studies have shown that this might be the case [63]–[67]. The existence of interference between transcription and replication is also supported by the observation that origins are often located in moderately expressed promoters but are excluded from very active promoters [23], and by the dramatic changes in the location of origins after the initiation of transcription during development of *Xenopus Laevis* [68] and mammalian cells [67].

An important question is: how are multi-megabases late domains replicated? These regions cannot be replicated passively because they are too far from early origins. The fewer origins of replication we observed in later stages of S-phase is consistent with the recent report that late replicating regions (including the inactive X chromosome) replicated faster than early regions and exhibit a random order of initiation [32]. This replication pattern might suggest either that replication in these regions does not initiate at consistent origins, or that replication initiation occurs synchronously at many concurrent origins. We detected a large number of low efficiency origins in these regions that are, similarly to early origins, located near G-quadruplexes, transcription start sites and CpG islands more often that would be expected by chance. We propose that large heterochromatic regions might be replicated from a subset of replication origins that are activated late in S phase and that can each replicate much larger regions than early origins because of the regular structure and lack of transcriptional activity of heterochromatin.

## Materials and Methods

### Ethics statement

This work was performed under approved Einstein IRB protocol number 2011-356-000.

### Cell culture

10 to 20 ml of peripheral white blood cells were harvested by venipuncture from individuals from family FNY01 and mononuclear cells were isolated by density gradient centrifugation on Histopaque (Sigma-Aldrich) according to the manufacturer's instructions. The purified cells were frozen in 2 million cell aliquots. Two million mononuclear cells were expanded and differentiated into basophilic erythroblasts in culture for two weeks in serum-free Stemspan media (Stem Cells Technologies, VA, CA) containing the cytokine cocktail mix described by Olivier et al. [37]. At the end of the culture, cells were immuno-phenotyped by FACS using antibodies against CD71 and CD235a. Cells were relatively uniform in size and more than 97% of the cells were double-positives demonstrating that the vast majority of cells in the culture were erythroid and at the basophilic stage of differentiation.

### Cell cycle analysis and S phase sorting

#### S phase DNA

Twenty to fifty million exponentially growing basophilic erythroblasts were harvested on day 14 of culture, washed with ice cold PBS, fixed by dropwise addition of ice-cold 75% ethanol, and stored at 4°C before processing. Fixed cells were treated with RNase A (0.5 mg/ml for 60 minutes, Sigma-Aldrich) and stained for DNA content with propidium iodide (50 µg/ml) (Invitrogen, Carlsbad, CA). Cells in the S phase of the cell cycle were sorted using a FACS Aria II (BD Biosciences). Genomic DNA was prepared using the Wizard Genomic DNA Purification Kit (Promega) and quantified using NanoDrop and PicoGreen methods (Quant-iT PicoGreen dsDNA Assay Kit, Invitrogen).

#### G1-phase DNA

Sequencing of family FNY01 to obtain the phased genomes was performed with DNA from peripheral blood white blood cells [36]. Since more than 99.5% of peripheral blood white blood cells are in the G1 phase of the cell cycle (Figure 1B), data from these experiments were used as the G1 control in all the TimEX-seq experiments described in this report.

### Pair-end library preparation

Genomic DNA was sheared to 300–600 bp size using a focused ultra-sonicator from Covaris (Woburn, MA), size purified by agarose gel electrophoresis, and analyzed on a DNA Chip using a 2100 bio-analyzer (Agilent Technologies, Inc.) to verify size distribution. DNA was end-repaired (End-It kit, Epicenter Biotechnologies), an A-overhang was added (Klenow 3′ to 5′ exo minus, NEB), and ligated to Illumina pair-end adapter sequences (Illumina). The ligated libraries were size selected (600±50 bp), PCR amplified for 10 cycles, and purified with SPRI beads (Agencourt AMPure XP; Fisher Scientific). Pair-end library quality was verified on a bio-analyzer as described above. Each library was sequenced on three lanes on the Illumina HiSeq 2000 yielding about 10^9^, one hundred bp pair-end reads corresponding to about 30× coverage for each S phase library.

### Generation of the allele specific TimEX profiles

After library sequencing, the reads were aligned to hg 19 with bwa [69] using the default parameters and the SNPs were called using the GATK variant caller in the known-allele mode [70], providing the vcf file that describes the genomes of family FNY01 [36] as a reference file. In this mode, the GATK calls only the SNPs at the positions specified in the user-provided reference vcf file.

Once the SNPs had been called, the vcf files for the S phase libraries was phased using the vcf for family FNY01 as a reference and specific functions in GenPlay multi-genome [71]. Once the files were phased files (in the BED format) containing the allele depth for the paternal and maternal chromosomes were generated, again using specific functions that are provided in GenPlay.

The read depth files were binned in to 500 bp windows, normalized to the total number of reads containing a SNP in each track and smoothed using the GenPlay Gaussian filter (sigma = 100 kb). S/G1 ratios for the paternal and maternal chromosomes were then calculated. The resulting numbers were multiplied by a factor 1.4 to take into account the fact that the average copy number in S is higher than in G1. These resulted in TimEX profiles with value ranging between 0.9 and 2.1. No other filter or normalization was applied to the data. Masks were created to eliminate the regions of low SNP-density using GenPlay island finder functions. The resulting masks were applied in selected analysis to ascertain that the results were independent of SNP density. Other smoothing techniques (Loess, moving average) yielded essentially the same results.

The combined FNY01_3_2&3_3 allele-specific replication profiles were produced by generating four new files: a combined G1 maternal file containing the sum of the allele-depths of the maternal homologs of FNY01_3_2 and 3_2; a combined G1 paternal file containing the sum of the allele depths of the paternal homologs of FNY01_3_2 and 3_2, and similarly, two combined maternal and paternal S files. These four files were then processed as above to generate combined maternal and paternal S/G1 profiles.

### Detection of asynchronously replicated domains

Asynchronously replicated regions were detected with the GenPlay island finder on a data file generated by subtracting the paternal S/G1 simulated profiles from the maternal S/G1 simulated profiles.

The GenPlay Island finder function is based on the SICER algorithm of Zang et al. [72] and identifies broad regions of enriched read counts rather than peaks. The algorithm allows for gaps in the island and is well suited for the allele-specific TimEX data because of the heterogeneous distribution of the SNPs in the human genome. Implementation of the algorithm in GenPlay is described in the GenPlay documentation in the GenPlay web site.

A TimEX difference threshold of 0.02, a maximum gap sizes of 250,000 and a minimum island size of 50,000 bp were used in the island finder. Statistically significant regions were then identified using a qui-square test for goodness of fit (chisq.test function in R) on 2×2 contingency tables built by calculating the total number of reads obtained in the S and G1 fraction of the maternal and paternal chromosomes for each islands. Q-values were then calculated using the p.adjust function in R (with the fdr parameter) to control for multiple testing. An FDR rate of 5% was used to define the statistically significant asynchronous replication domains. The parameters for the island finder were determined by optimizing by trial and error, the rate of discovery of simulated regions (see below).

Core detection: Core were detected as ARD except that a TimEX difference threshold of 0.1, a maximum gap sizes of 50,000 and a minimum island size of 50,000 bp were used in the GenPlay Island finder. Statistical significance was determined as above.

### TimEX simulations

Simulated maternal and paternal pairs of S and G1 resampled control profiles were generated by imputing in a random number generator based on the binomial function (similar to the rbinom function in R), the number of S reads and the total number of reads (S+G1 reads) observed for each informative SNP in the maternal track of FNY01_3_2.

About 1,000 randomly located simulated asynchronous genomic regions (ranging in size from 125,000 to 2,000,000 bp) were then introduced in the simulated maternal control S profiles by increasing by a fixed percentage (5, 10, 15, 20, 30 or 50%), the total (S+G1) number of reads observed for each SNP and by assigning the extra reads to the S phase profile. These S and S+G1 read numbers were then imputed in the random generator to generate resampled read numbers in these simulated asynchronous regions.

Once simulated number of reads for the S and G1 profiles of the maternal and paternal homologs had been generated, simulated S/G1 TimEX profiles were generated as described above using Gaussian smoothing (sigma = 100 kb). Bedgraph files were generated to visualize the simulation in GenPlay.

Asynchronously replicated regions in these simulated profiles were then detected with GenPlay island finder as described above. Simulations were repeated multiple times.

A Java script implementing this algorithm can be found at https://github.com/JulienLajugie/ReplicationTimingSimulation.

### Generation of non-allele specific hi-res TimEX profiles

S and G1 aligned reads obtained after alignment to hg19 were loaded into 1 kb bins in GenPlay, normalized to the total number of reads, S to G1 ratios were calculated, outliers were eliminated (S/G1>2.4), and the profiles were smoothed with the GenPlay Gaussian filter (sigma = 20 kb). Use of other smoothing techniques (Loess, moving average) yielded essentially the same results. For more convenient analysis values were then indexed between 1 and 2.

### Nascent strands

Nascent, newly replicated DNA strands from cultured basophilic erythroblasts were isolated following two protocols. First, we have isolated RNA-primed short newly replicated DNA following the procedure described in Martin et al. [23]. Briefly, DNA was extracted from asynchronous cultures, denatured and fractionated by centrifugation on a neutral sucrose gradient. DNA fractions within the 0.5 kb to 1 kb size range were collected and DNA was exposed to lambda exonuclease to remove non-RNA-primed genomic DNA fragments. Purified RNA-primed NS were random-primed and the resulting double-stranded nascent DNA (1 µg) was sequenced using the Illumina (Solexa) genome analyzer II.

Second, to control for possible lambda exonuclease biases, we have also isolated and sequenced short nascent DNA strands that were isolated based on incorporation of the nucleotide analog BrdU as described by Aladjem et al. [40]. Briefly, we labeled cells with a 30-minute pulse of BrdU, lysed the cells and fractionated small DNA strands (<1 kb) using a neutral sucrose gradient. We then employed immune-precipitation with anti-BrdU antibodies to isolate newly replicated DNA strands on the basis of selective incorporation of BrdU. The resulting NS were subject to massively parallel sequencing. For both methods, sheared genomic DNA was sequenced as a standard to control for mapability and other potential biases.

Nascent libraries were prepared and sequenced on two lanes of an Illumina GII analyzer. Read length was 36 bp. Reads were aligned to hg19 using bwa and loaded in GenPlay in bins of 100 bp, 3 kb or 20 kb (depending on the analysis). Peaks were called using MACS 1.42 using the default parameters, or the GenPlay Island finder.

### Analysis of the effect of structural variants on timing of replication

The ratios of the TimEX values of the two chromosome homologs for individuals FNY01 3_2 and 3_3 were calculated in 5 kb windows and averaged over 500 kb intervals (overlapping every 100 kb). Ratios smaller than 1 were inverted, since we expected the SVs to either increase or decrease the timing ratios. SVs present in the family are described in detail in [36].

Similar results were obtained with interval of 1 Mb, with non-overlapping intervals of 0.5 or 1 Mb, or by averaging the distance or the absolute values of the differences between the S/G1 ratios of the homologs rather than their ratios.

### Correlation between hi-res timing profiles and NS DNA

Hi-res TimEX profiles of FNY01 3_2 and 3_3 were averaged and binned at 3 kb. NS were binned at 200 bp and smoothed with the GenPlay Gaussian filter using sigma 3 kb or 20 kb. The tracks were then converted to 3 kb bins after saturating the outliers (i.e. all bins with scores larger than X with x = mean plus 3 times the standard deviation were set to a score equal to X). The averaged TimEX profiles were then divided into 5 equal fractions termed S1 to S5 based on the TimEX values (with S1 replicating the earliest and S5 the latest). Correlation and linear regression were performed in R or in Statgraphics Centurion XVII (Statpoint Technologies incorporated). Removing the outliers rather than saturating them yielded similar results.

### Calling of the NS peaks

Peaks were called using MACS v1.42 using the default value (p<10E-5 and FDR<0.2%). Sheared genomic DNA from the same individual was used as the control.

### Comparison of the BrdU and lambda exonuclease method and association with G-quadruplexes, CpG islands, transcription start sites and DNase I hypersensitivity

The number of peaks observed with the BrdU and lambda exonuclease methods was similar (respectively 250,000 and 250,00), the average widths of the peaks were also similar, about 100–400 bp in both cases. To facilitate the comparison between the methods, all peaks were normalized to a width of 400 bp which resulted in a 50% reduction in peak number and the analysis was performed on the top 100,000 peaks only.

Peaks were considered to be associated to a genomic features if they overlapping by one base pair or more with a CpG Island, a transcription start site (defined as 1 kb fragment centered on the start site) or with 400 bp fragments centered around a G-quadruplexes or a DNase I hypersensitive site.

### GC content

GC content was calculated as the average percent of C and G in 100 kb windows (in 10 kb intervals).

### Calling of the summits of the TimEX ripples and of the NS clusters

Because of the very uneven baseline, MACS or the Genplay island finders were not able to satisfactorily detect and call the ripples within the larger TimEX domains that are illustrated in Figure 4. To call these peaks we therefore devised a specific strategy illustrated in Figure S5. Briefly, the TimEX profiles were smoothed with the Gaussian filters and a sigma at 20 kb (which revealed the ripples) and at 100 kb (which smooth out these ripples) and the 100 kb smooth was subtracted from the 20 kb smooth. The top 10 percent of the peaks thus obtained were then analyzed since they coincided closely with the major ripples that we wished to characterize. The same strategy was used to define the clusters of NS.

### Statistics

All statistical analysis were performed using R or using StatGraphics Centurion XVI (Statpoint technologies)

### GenPlay multi-genome

Most of the data processing and visualization was performed in GenPlay multi-Genome, an application that was written to display, produce and process allele-specific and non-allele-specific sequencing data files. GenPlay is available freely at http://genplay.einstein.yu.edu.

### Data

#### Imprinted genes

The list of known and predicted genes was obtained from the geneimprint.com web site. The list is reproduced in Table S3.

#### G-quadruplexes

Location of G-quadruplexes was generously provided by Dr. Julian Huppert and is described in [73]. Data was converted to hg19 coordinate using liftover.

#### DNase I hypersensitivity data

DNase I hypersensitivity data from Fetal Liver erythroid cells and K562 was generously provided by Dr. John Stamatoyannopoulos (University of Washington Seattle). It is available from the UCSC browser.

#### CpG islands, RefSeq gene annotations and repeat data

Annotations were downloaded from the UCSC genome browser on January 2013.

#### Repeat maps

Repeats map were downloaded on January 213 from UCSC and were originally from repbase.

#### Accession number

Processed and raw data files have been deposited at NCBI in the Geo database. Accession number: GSE50978

#### GenPlay project

The entire dataset can be visualized in the interactive GenPlay environment at http://genplay.einstein.yu.edu/library/projects/TimEX-NascentStrands-2013/.

## Supporting Information

Figure S1 Normality plots of the differences of TimEX values for the maternal and paternal homologs. Red curve: ideal normality plot. Blue curve: observed normality of the differences in TimEX values between the maternal and paternal homologs. Deviation from normality suggests the existence of regions with differential timing between the two homologs.
(TIF)

Figure S2 Allele-specific TimEX-seq. A: Comparison of the timing of replication in genetically identical and non-identical regions. The coefficients of correlation between either the timing of replication of the two maternally inherited, or the two paternally inherited chromosomes, was calculated in 500 kb windows in the identical and non-identical regions of the genome of FNY01 3_2 and 3_3. The timing of replication is very similar in the identical and non-identical regions. B: Box and whiskers plots illustrating the distribution of the differences of the mean-centered TimEX values of the maternal homologs of individuals FNY01_3_2 and 3_3 in the identical (ID) and non-identical (Non ID) regions. Differences were calculated in 5 kb (left) or 500 kb (right) windows. The timing profiles in the identical and non-identical regions are very similar. Similar results were observed when the paternal homologs were compared. C: SVs can cause replication asynchrony: Box-and-whisker plots illustrating the distribution of the timing of replication differential of the paternal and maternal homologs in 500 kb intervals containing either no SV (No SV), or SVs larger than 10, 50 AND 100 kb for individual FNY01_3_2 and 3_3. The boxes represent the middle 50% of the data; the vertical lines, the median; the brown cross the mean, the mean; the whiskers; the middle 75% of the data; and the squares, the outliers. For each 500 kb interval, the timing differential was calculated as the average of the S/G1 ratios computed in 500 kb windows. Ratios smaller than one were inverted to capture the absolute value of the timing differential. D: Comparisons of the means of the allelic timing ratios in 500 kb windows in individual FNY01 3_2 and 3_3 using Fisher's least significant difference (LSD) procedure. Presence of SVs increases the allelic TimEX ratios when compared with 500 Mb regions that do not contain SVs. Similar results were obtained using windows of 1 Mb, using overlapping or non-overlapping intervals, and calculating the timing differential as log ratio, or as distance between the allelic TimEX values.
(TIF)

Figure S3 Detection of Asynchronously Replicated Domains. Simulated regions of asynchrony were introduced into the data and an automated method to identify statistically significant Asynchronously Replicated Domains was developed (see methods). The top graphs illustrate the sensitivity of allele-specific TimEX-seq for simulated asynchronous regions of different sizes and levels of asynchrony. The Y-axis represents the detection rate calculated as the ratio of the number of detected islands overlapping with simulated islands to the total number of simulated islands introduced in the data. The X-axis represents the delays (in minutes) simulated in the data. Each bar in the histograms represents the results for about 700 islands introduced at randomly position in the genome. Repeat of these simulations gave very similar results. Second graph from the top illustrates the sensitivity of Allele-specificTimEX-seq. The false discovery rate (specificity) was calculate as the number of statistically significant island detected that did not overlap with simulated islands to the number of detected island that did overlap with simulated islands introduced in the data. The observed false discovery rates were in excellent agreement with the theoretical 5% discovery rate use to adjust the p-value to correct for multiple testing during island detection. The bottom two graphs illustrate the size and timing differentials caused by our data processing. The distortion in the amplitude of the timing differential was calculated as the ratio of the observed ARD average timing differential to the simulated ARD average timing differential. The distortion in the size of the ARD was calculated as the ratio of the observed ARD size to the simulated ARD size. Errors bars represent the standard error of the mean. Since the rate of detection was very high and since size and amplitude distortions was relatively minor for islands larger than 500,000, we limited our study to island at least 500,000 bases in size.
(TIF)

Figure S4 Association of BrdU NS peaks with G-quadruplexes, transcription starts sites and CpG Islands. A: Pie-chart summarizing the number of BrdU NS peaks associated with G-quadruplexes, transcription start sites, and CpG Islands. B: Most prototypical origins contain at least one of these three genomic features. C: Chromatin accessibility favors the formation of origins of replication at G-quadruplexes, transcription start sites and CpG islands. Red bars represent the percent of G-quadruplexes, transcription start sites and CpG islands that are associated with a BrdU NS peaks. Green and blue bars respectively represent the percent of G-quadruplexes, transcription start sites and CpG islands that are located (green) and are not located (blue) within a DNase I hypersensitive site and that are associated with a BrdU NS peaks. Fetal liver basophilic erythroblasts DNase I hypersensitivity data was obtained from the Stammayanopoulos lab. D: Proportion of G-quadruplexes, CpG islands and transcription start sites that are DNase I hypersensitive in K562 cells and in fetal liver erythroid cells. Only a few percent of the BU NS peaks, G-quadruplexes, CpG Islands and transcription start sites are DNase I hypersensitive sites.
(TIF)

Figure S5 TimEX peaks and BrdU NS co-localize. A: Left panels. Top: Hi-Res TimEX profile for a 2.5 million base pair region on chr.14 (20 kb Gaussian smooth). Middle: red curve: TimEX profile for same region, Gaussian smooth (sigma = 100 kb). Solid blue curve: TimEX Gaussian smooth (same as above, sigma = 20 kb). Bottom: Difference between the 20 and 100 kb smooths yields the small TimEX peaks. Right panels: Top: Profiles of NS distribution at 100 bp resolution. Middle: solid green curve: Gaussian smooth (sigma = 20 kb) of BrdU NS. Red curve: Gaussian smooth (sigma = 100 kb) of BrdU NS. Bottom: Difference between the 20 and 100 kb smooths yields the BrdU clusters. Peaks and clusters were defined with this Gaussian smooth differential method because classical peak callers such as MACS or the GenPlay peak finders could not efficiently resolve the TimEX peaks due to the highly variable baseline. The top 10% of the TimEX peaks (by area) closely match the peaks and valleys within the larger timing domains and were compared to the top 10% of the BrdU NS clusters. B: Tables summarizing the overlap between the TimEX and BrdU peaks. TimEX profiles were computationally divided in 5 S phase fractions (S1 to S5, with S1 earliest fraction) and the number of TimEX peaks, the number of overlapping BrdU clusters and the percent overlap were calculated for each fraction. The S1 fraction contains the largest number of peaks and 68% of the TimEX peaks overlapped with a BrdU NS cluster. This was highly statistically significant because the TimEX peaks and the BrdU clusters respectively covered about 21.9% and 18.4% of the DNA replicated in S1. It therefore follows that the probability of random overlap can be computed as 0.219 * (3* 0.184) = 0.119 or 11.9%. A factor three is included in the calculation to take in consideration partial overlaps. Randomization analysis confirmed this analysis since 100 randomized simulation of the data yielded an average overlap of 11.9%.
(TIF)

Table S1 Number of allele specific uniquely mapped reads obtained after sequencing individual FNY01 3_2 and 3_3.
(XLSX)

Table S2 Statistically significant regions in FNY01 3_2 (A), 3_3 (B) or the combined 3_2 and 3_3 tracks (C). Asynchronously replicated Domains (ARD) and core ARD were defined as discussed in the text. Imprinted genes and imprinted status are from geneimprint.com. When multiple genes are present in the same regions, only one of the gene is shown. Calculations for the p and q-values is described in the text. Coverage: Fraction of 500 bp windows in the ARD that contained at least one SNP. ARD Delay: Average of the differences of the S/G1 copy number ratio from the maternal and paternal homologs multiple by 480 (which is the length of the S phase in minutes). A factor 1.2 was applied to take into consideration the fact that the TimEX value ranged from 0.9 to 2.1 rather that the theoretical 1 to 2 Length (core): length of the core in bases (or sum of the length of the core when ARD contain more than one core). Delay (core): same calculation as ARD delay but taking into consideration the windows present in the core.
(XLSX)

Table S3 List of imprinted genes and their timing asynchrony in erythroblasts. See text for definition of ARD, core ARD and method to calculate the delays. Some ARD exhibited several cores other did not have any detectable core with the parameter used in the island finder.
(XLSX)

Table S4 Coefficient of correlation between TimEX-seq and BrdU nascent strand profiles. S/G1 ratios smoothed at 20 kb and BrdU NS density were compared genome-wide. Both the S/G1 ratio and the BrdU NS were binned into 3 Kb windows. The S/G1 ratio was smoothed using a 20 kb sigma. The BrdU NS were either unsmoothed or smoothed with a 20 kb sigma.
(XLSX)
