# Supplementary figures and images for: Allele-Specific Genome-wide Profiling in Human Primary Erythroblasts Reveal Replication Program Organization
Source: PLoS Genet. 2014 May 1;10(5):e1004319. doi: 10.1371/journal.pgen.1004319 (PMC4006724; doi:10.1371/journal.pgen.1004319)

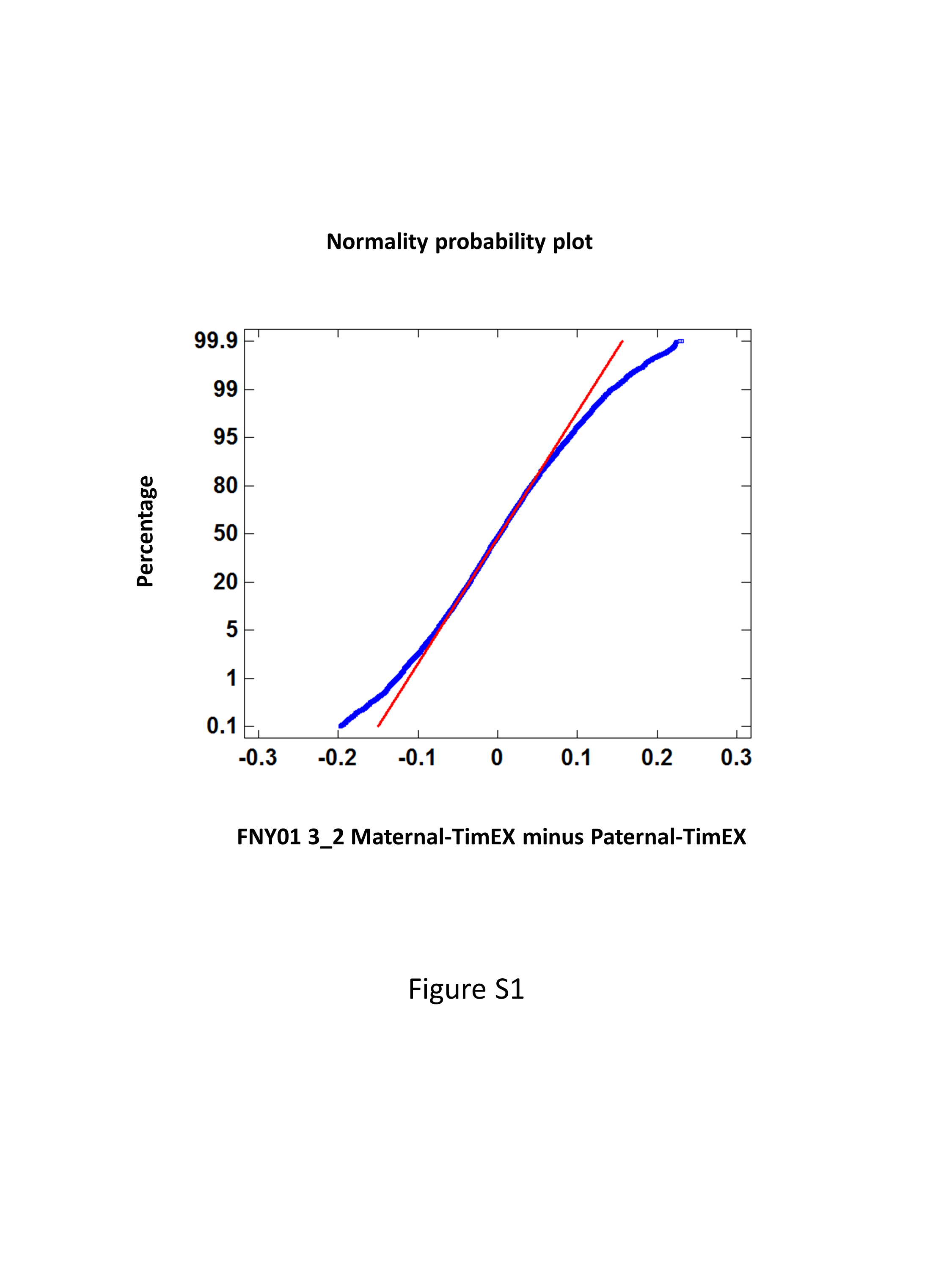

Supplement: Figure S1 — Normality plots of the differences of TimEX values for the maternal and paternal homologs. Red curve: ideal normality plot. Blue curve: observed normality of the differences in TimEX values between the maternal and paternal homologs. Deviation from normality suggests the existence of regions with differential timing between the two homologs. (TIF) [file pgen.1004319.s001.tif]

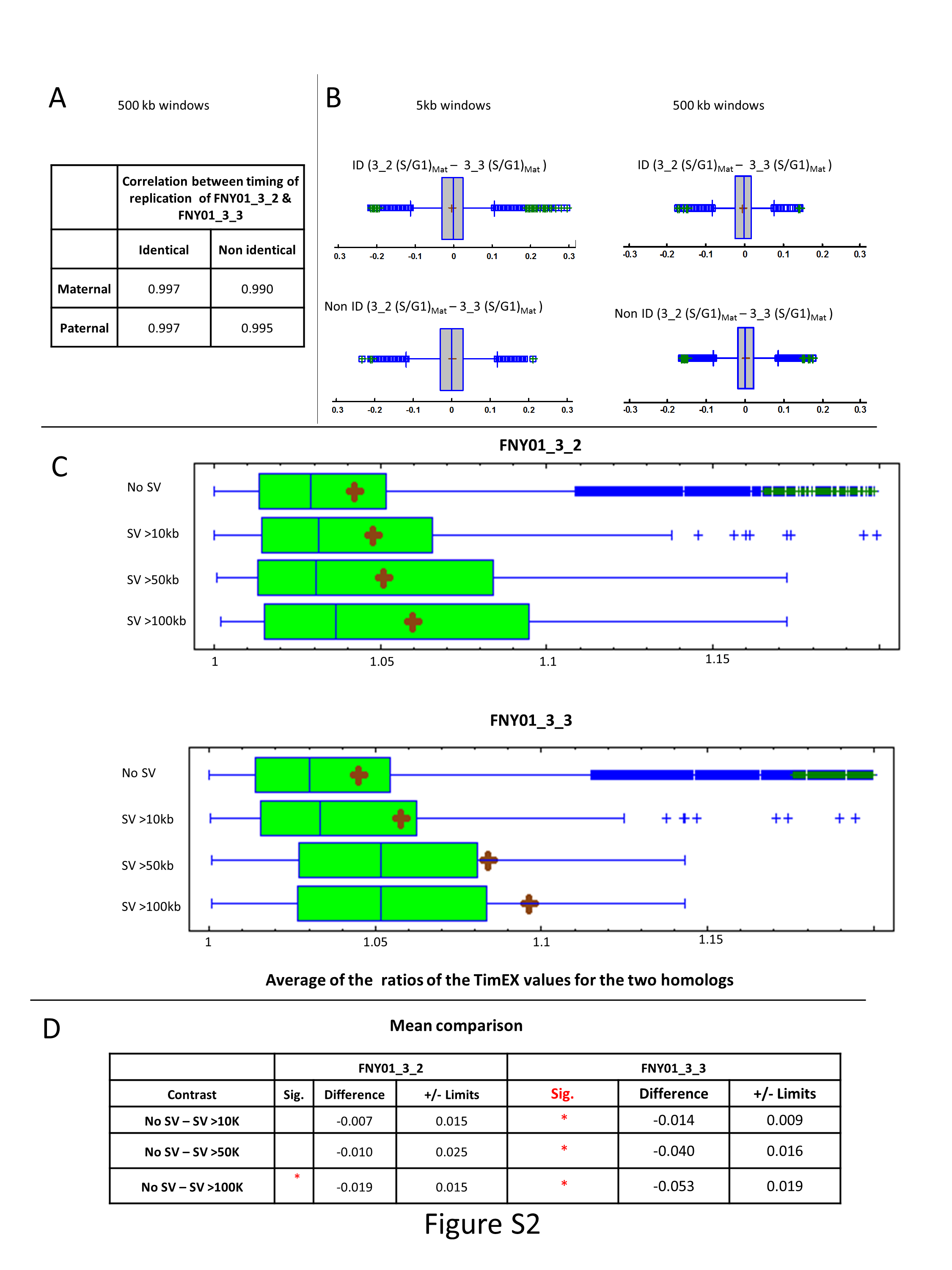

Supplement: Figure S2 — Allele-specific TimEX-seq. A: Comparison of the timing of replication in genetically identical and non-identical regions. The coefficients of correlation between either the timing of replication of the two maternally inherited, or the two paternally inherited chromosomes, was calculated in 500 kb windows in the identical and non-identical regions of the genome of FNY01 3_2 and 3_3. The timing of replication is very similar in the identical and non-identical regions. B: Box and whiskers plots illustrating the distribution of the differences of the mean-centered TimEX values of the maternal homologs of individuals FNY01_3_2 and 3_3 in the identical (ID) and non-identical (Non ID) regions. Differences were calculated in 5 kb (left) or 500 kb (right) windows. The timing profiles in the identical and non-identical regions are very similar. Similar results were observed when the paternal homologs were compared. C: SVs can cause replication asynchrony: Box-and-whisker plots illustrating the distribution of the timing of replication differential of the paternal and maternal homologs in 500 kb intervals containing either no SV (No SV), or SVs larger than 10, 50 AND 100 kb for individual FNY01_3_2 and 3_3. The boxes represent the middle 50% of the data; the vertical lines, the median; the brown cross the mean, the mean; the whiskers; the middle 75% of the data; and the squares, the outliers. For each 500 kb interval, the timing differential was calculated as the average of the S/G1 ratios computed in 500 kb windows. Ratios smaller than one were inverted to capture the absolute value of the timing differential. D: Comparisons of the means of the allelic timing ratios in 500 kb windows in individual FNY01 3_2 and 3_3 using Fisher's least significant difference (LSD) procedure. Presence of SVs increases the allelic TimEX ratios when compared with 500 Mb regions that do not contain SVs. Similar results were obtained using windows of 1 Mb, using overlapping or non-overlapping inte [file pgen.1004319.s002.tif]

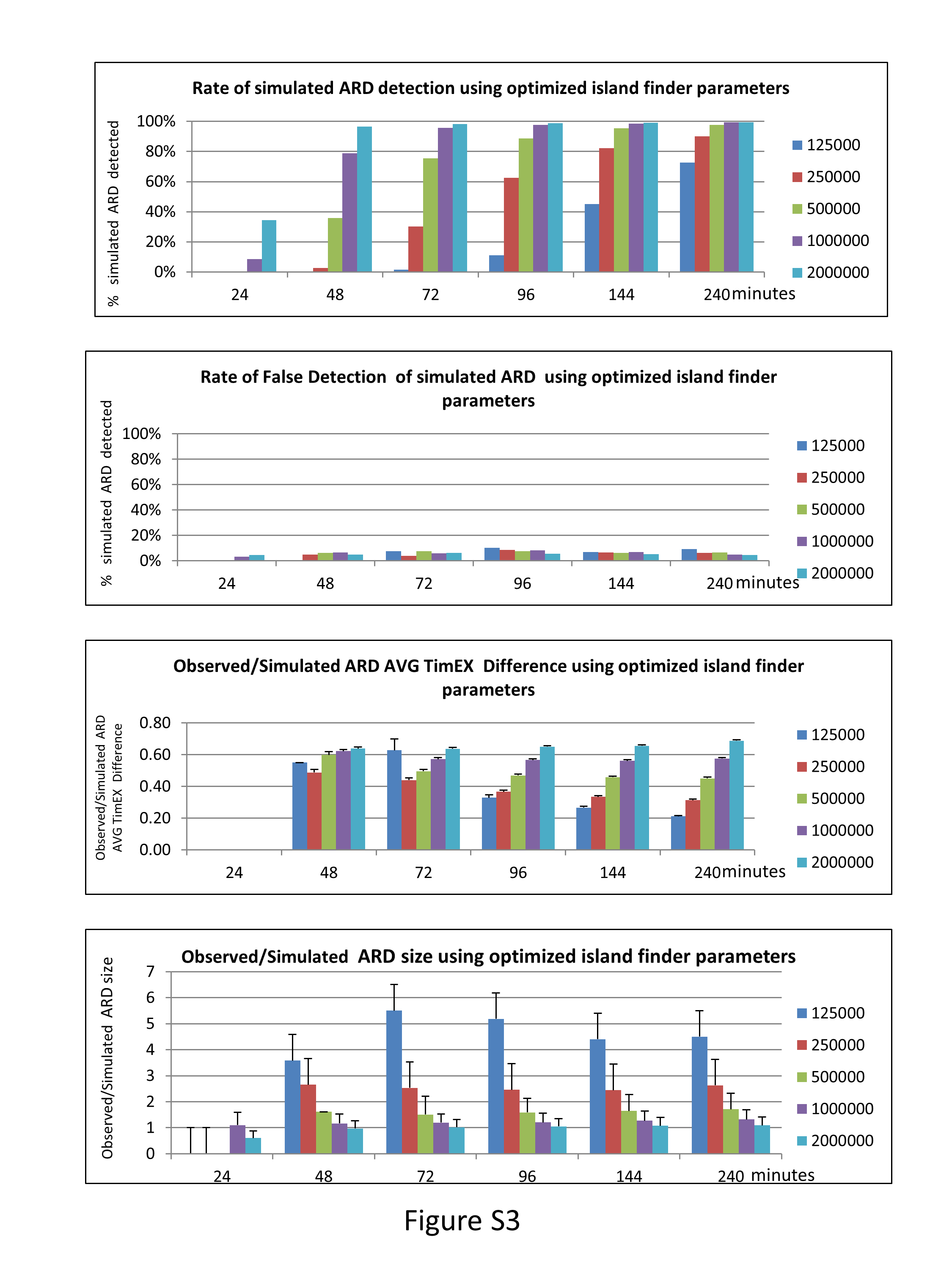

Supplement: Figure S3 — Detection of Asynchronously Replicated Domains. Simulated regions of asynchrony were introduced into the data and an automated method to identify statistically significant Asynchronously Replicated Domains was developed (see methods). The top graphs illustrate the sensitivity of allele-specific TimEX-seq for simulated asynchronous regions of different sizes and levels of asynchrony. The Y-axis represents the detection rate calculated as the ratio of the number of detected islands overlapping with simulated islands to the total number of simulated islands introduced in the data. The X-axis represents the delays (in minutes) simulated in the data. Each bar in the histograms represents the results for about 700 islands introduced at randomly position in the genome. Repeat of these simulations gave very similar results. Second graph from the top illustrates the sensitivity of Allele-specificTimEX-seq. The false discovery rate (specificity) was calculate as the number of statistically significant island detected that did not overlap with simulated islands to the number of detected island that did overlap with simulated islands introduced in the data. The observed false discovery rates were in excellent agreement with the theoretical 5% discovery rate use to adjust the p-value to correct for multiple testing during island detection. The bottom two graphs illustrate the size and timing differentials caused by our data processing. The distortion in the amplitude of the timing differential was calculated as the ratio of the observed ARD average timing differential to the simulated ARD average timing differential. The distortion in the size of the ARD was calculated as the ratio of the observed ARD size to the simulated ARD size. Errors bars represent the standard error of the mean. Since the rate of detection was very high and since size and amplitude distortions was relatively minor for islands larger than 500,000, we limited our study to island at least 500,000 bases in si [file pgen.1004319.s003.tif]

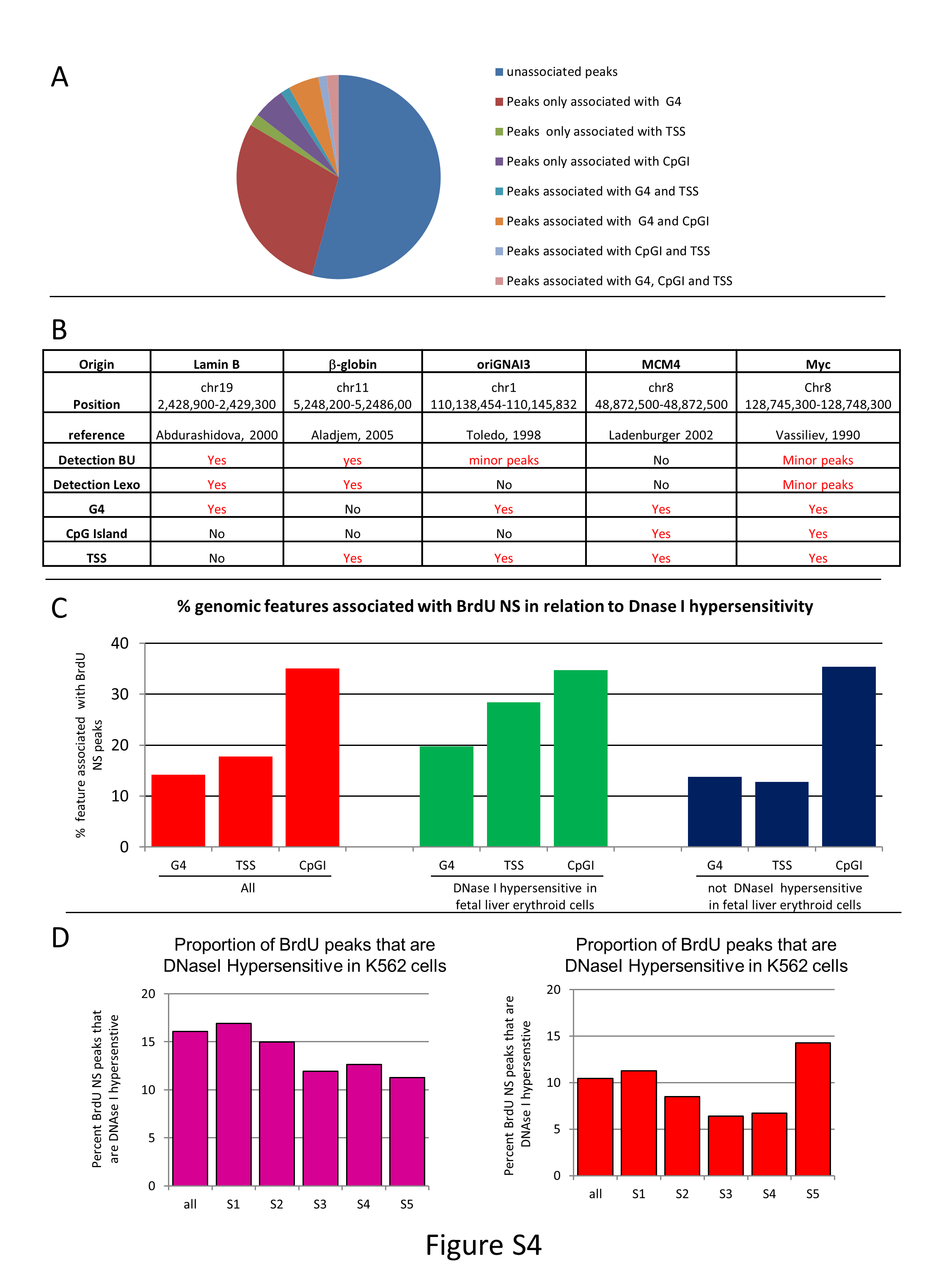

Supplement: Figure S4 — Association of BrdU NS peaks with G-quadruplexes, transcription starts sites and CpG Islands. A: Pie-chart summarizing the number of BrdU NS peaks associated with G-quadruplexes, transcription start sites, and CpG Islands. B: Most prototypical origins contain at least one of these three genomic features. C: Chromatin accessibility favors the formation of origins of replication at G-quadruplexes, transcription start sites and CpG islands. Red bars represent the percent of G-quadruplexes, transcription start sites and CpG islands that are associated with a BrdU NS peaks. Green and blue bars respectively represent the percent of G-quadruplexes, transcription start sites and CpG islands that are located (green) and are not located (blue) within a DNase I hypersensitive site and that are associated with a BrdU NS peaks. Fetal liver basophilic erythroblasts DNase I hypersensitivity data was obtained from the Stammayanopoulos lab. D: Proportion of G-quadruplexes, CpG islands and transcription start sites that are DNase I hypersensitive in K562 cells and in fetal liver erythroid cells. Only a few percent of the BU NS peaks, G-quadruplexes, CpG Islands and transcription start sites are DNase I hypersensitive sites. (TIF) [file pgen.1004319.s004.tif]

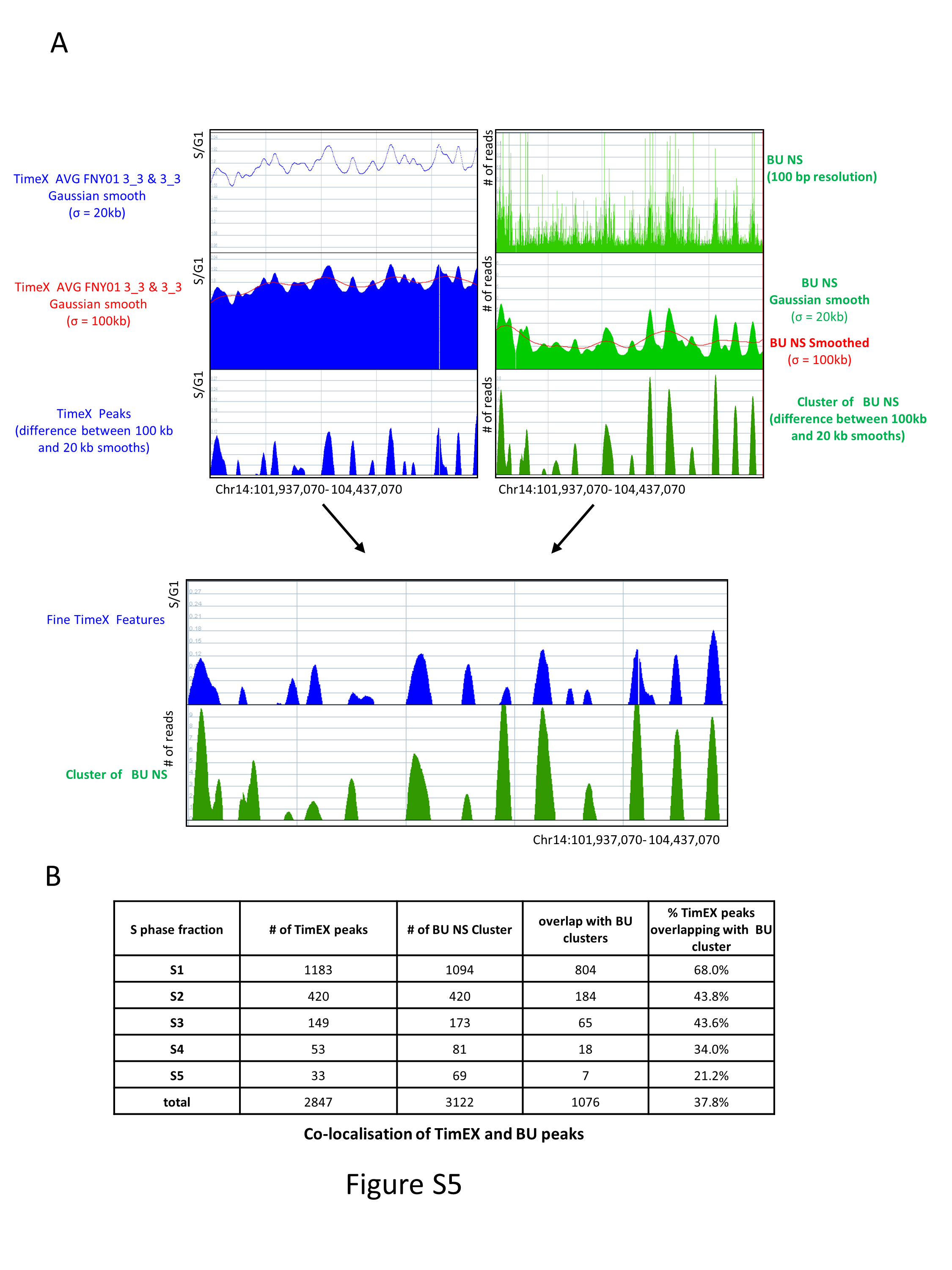

Supplement: Figure S5 — TimEX peaks and BrdU NS co-localize. A: Left panels. Top: Hi-Res TimEX profile for a 2.5 million base pair region on chr.14 (20 kb Gaussian smooth). Middle: red curve: TimEX profile for same region, Gaussian smooth (sigma = 100 kb). Solid blue curve: TimEX Gaussian smooth (same as above, sigma = 20 kb). Bottom: Difference between the 20 and 100 kb smooths yields the small TimEX peaks. Right panels: Top: Profiles of NS distribution at 100 bp resolution. Middle: solid green curve: Gaussian smooth (sigma = 20 kb) of BrdU NS. Red curve: Gaussian smooth (sigma = 100 kb) of BrdU NS. Bottom: Difference between the 20 and 100 kb smooths yields the BrdU clusters. Peaks and clusters were defined with this Gaussian smooth differential method because classical peak callers such as MACS or the GenPlay peak finders could not efficiently resolve the TimEX peaks due to the highly variable baseline. The top 10% of the TimEX peaks (by area) closely match the peaks and valleys within the larger timing domains and were compared to the top 10% of the BrdU NS clusters. B: Tables summarizing the overlap between the TimEX and BrdU peaks. TimEX profiles were computationally divided in 5 S phase fractions (S1 to S5, with S1 earliest fraction) and the number of TimEX peaks, the number of overlapping BrdU clusters and the percent overlap were calculated for each fraction. The S1 fraction contains the largest number of peaks and 68% of the TimEX peaks overlapped with a BrdU NS cluster. This was highly statistically significant because the TimEX peaks and the BrdU clusters respectively covered about 21.9% and 18.4% of the DNA replicated in S1. It therefore follows that the probability of random overlap can be computed as 0.219 * (3* 0.184) = 0.119 or 11.9%. A factor three is included in the calculation to take in consideration partial overlaps. Randomization analysis confirmed this analysis since 100 randomized simulation of the data yielded an average overlap of 11.9%. (TIF) [file pgen.1004319.s005.tif]
